# Genes Selectively Expressed in Rat Organs

**DOI:** 10.2174/0113892029273121240401060228

**Published:** 2024-04-08

**Authors:** Dan Li, Xulian Wan, Yu Yun, Yongkun Li, Weigang Duan

**Affiliations:** 1 The Department of Pharmacology, School of Basic Medicine, Kunming Medical University, Kunming, 650500, China;; 2 School of Basic Medicine, Yunnan University of Traditional Chinese Medicine, Kunming, 650500, China

**Keywords:** High-throughput sequencing, selective expression, organic markers, rat, genetic variations, DNA

## Abstract

**Background:**

Understanding organic functions at a molecular level is important for scientists to unveil the disease mechanism and to develop diagnostic or therapeutic methods.

**Aims:**

The present study tried to find genes selectively expressed in 11 rat organs, including the adrenal gland, brain, colon, duodenum, heart, ileum, kidney, liver, lung, spleen, and stomach.

**Materials and Methods:**

Three normal male Sprague-Dawley (SD) rats were anesthetized, their organs mentioned above were harvested, and RNA in the fresh organs was extracted. Purified RNA was reversely transcribed and sequenced using the Solexa high-throughput sequencing technique. The abundance of a gene was measured by the expected value of fragments per kilobase of transcript sequence per million base pairs sequenced (FPKM). Genes in organs with the highest expression level were sought out and compared with their median value in organs. If a gene in the highest expressed organ was significantly different (*p* < 0.05) from that in the medianly expressed organ, accompanied by q value < 0.05, and accounted for more than 70% of the total abundance, the gene was assumed as the selective gene in the organ.

**Results & Discussion:**

The Kyoto Encyclopedia of Genes and Genomes (KEGG), and Gene Ontology (GO) pathways were enriched by the highest expressed genes. Based on the criterion, 1,406 selective genes were screened out, 1,283 of which were described in the gene bank and 123 of which were waiting to be described. KEGG and GO pathways in the organs were partly confirmed by the known understandings and a good portion of the pathways needed further investigation.

**Conclusion:**

The novel selective genes and organic functional pathways are useful for scientists to unveil the mechanisms of the organs at the molecular level, and the selective genes’ products are candidate disease markers for organs.

## 1 INTRODUCTION

It was once believed that all somatic cells shared the same genome because all of a creature's cells and organs develop from a fertilized egg. The expression of an animal’s genome controls the animal’s functions, whose functions are executed by its cells. Therefore, cells have different functions depending on different gene expression profiles [1, 2], and so do different tissues and organs. The other gene expression profiles will doom cell differentiation [3], organ development [4], and its functions. Based on the understanding, it can be assumed that some genes as constructive ones must be universally expressed in all the cells with a nucleus, and some could be selectively expressed in cells, tissues, and organs at different developmental stages [5, 6]. At an animal’s adulthood, its gene expression profiles could be relatively stable to maintain its biological functions, and the gene expression profile would reflect its function. Therefore, the products (RNAs and proteins) from the gene selectively expressed in an organ suggest its function(s).

Health and disease are the eternal themes of humans, and are usually related to gene expression profiles. The mechanism study on human health and disease is generally carried on model animals at first, then on humans. Among them, adult rats and mice are model animals most frequently used by scientists, and no animals are studied more deeply than them. Therefore, it is a good strategy to understand humans by investigating gene expression profiles in rats. Identifying molecular targets and disease markers from rats and mice is usually the first step to understanding human health and disease, then to finding therapeutic strategies and methods. The selective gene products released into the blood can be used as damage markers. However, it is a big premise to understand the normal model animal’s biological features at the molecular level before scientists comprehensively understand human health and disease [7]. There were much data from animals suggesting that some genes selectively expressed in organs, *e.g*. NeuN (Rbfox3) in the brain or neuron [8] though with alternative opinions [9], troponin (Tnnc1, Tnni3) in the heart [10], glutamic pyruvic transaminase (GPT, Gpt) in the liver [11], and neutrophil gelatinase-associated lipocalin (NGAL) in the kidney [12]. The findings are very useful and even were adopted for clinic diagnosis and treatment. The gene products selectively and originally distributed can be used as molecular organic markers and then make disease diagnosis more accurate or earlier. Nevertheless, in the background of precision medicine [13], the selective gene products in organs are still insufficient for clinical practice, and it is still necessary to systematically screen the genes selectively expressed in organs.

Proteins and RNAs are the end products of genes and execute their functions. To identify the selective functions at the molecular level, all the selectively distributed proteins in organs should be screened out. However, among them, protein screening is a big economic burden because the study would consume plenty of antibodies. Since proteins and RNAs were transcribed and even then translated from genes, the present study would apply high-throughput sequencing technology to analyze gene expression profiles of 11 organs, including the adrenal gland, brain, colon, duodenum, heart, ileum, kidney, liver, lung, spleen, and stomach, at the RNA level, and then, based on the results, to find the likely organic markers and analyze the functional pathways the selective genes would be involved in.

## 2 MATERIALS AND METHODS

### 2.1 Materials

Adult male Sprague-Dawley (SD) rats (age, 45 days; body weight, 180-220 g) were obtained from Chengdu Dossy Experimental Animal Co. Ltd., Chengdu, China [Certification No. SCXK (Chuan) 2008–24]. TRIzol Plus RNA Purification kit was purchased from Invitrogen (Carlsbad, CA, USA). Ultra-pure water was produced with a Milli Q water purification system manufactured by EMD Millipore Group (Darmstadt, Germany). NanoDrop ND-1000 spectrophotometer was manufactured by PeqLab (Erlangen, Germany). The multimicroplate reader of Infinite 200pro was manufactured by Tecan Group (Mannedorf, Switzerland). Other instruments or reagents used in the present study were made in China if not mentioned.

### 2.2 Animal Treatment

Three rats were normally treated for three days. Then, the animals were intraperitoneally anesthetized with urethane (1.0 g/kg). The rats’ chests and abdomens were opened, and their organs were harvested, including the adrenal gland (Ad), brain (frontal cortex) (Br), colon (Co), duodenum (the first 5 cm) (Du), heart (left ventricle) (He), ileum (the end 5 cm) (Il), kidney (right) (Ki), liver (Li), lung (right) (Lu), spleen (Sp), and stomach (gastric antrum) (St). The tunica and mesentery of the organs were removed clearly. All the organs were frozen with liquid nitrogen and kept at -80°C by dry ice to keep them fresh, and then sent to Sangon Biotech Co. Ltd. (Shanghai China) (https://www.sangon.com/) immediately for high-throughput sequencing.

The animal experiments were approved by the Animal Care and Use Committee of Yunnan Provincial Key Laboratory of Molecular Biology for Sinomedicine (Approved No. LL-20171023-01), Yunnan University of Traditional Chinese Medicine.

### 2.3 High-throughput Sequencing of mRNA

The fresh organs were frozen with liquid nitrogen and ground to powder. The total RNA in the powder was extracted and purified using the TRIzol Plus RNA Purification kit (Invitrogen, Carlsbad, CA, USA). The quantity and quality of RNA were measured by the NanoDrop ND-1000 spectrophotometer. RNA integrity was assessed by three bands (28S, 18S, and 5S) using formaldehyde denaturing agarose gel electrophoresis RNA as previously described [14, 15].

Similar to the results of our previous study [16], double-stranded cDNA (ds-cDNA) was reversely transcribed from the total RNA using a SuperScript ds-cDNA synthesis kit (Invitrogen, Carlsbad, USA) in the presence of 100 pmol/L oligo dT primers. Solexa high-throughput sequencing technique was used to sequence the cDNA by Sangon Biotech Co. Ltd. (Shanghai, China). The raw data containing reads of 150 bases of nucleotide in fastq format was transformed to original sequences in fasta format by Seqkit software in the disc operation system (DOS) model [17]. The sequences that matched 27 bp or more to the rat’s reference mRNA sequences (https://www.ncbi.nlm.nih.gov/) were screened out by TBtools software (v0.664445552). The expected value of fragments per kilobase of transcript sequence per million base pairs sequenced (FPKM) was used for the normalization of expression level [18].

### 2.4 Screening Genes Selectively Expressed

Values of gene’s FPKM in every organ were collected. The overall function of the organs at the gene expression level was analyzed by cluster analysis. The distance between organs was calculated by the Vegan package of Bray curtis method [19], and the cluster tree was established by Hcluster [20].

Based on the assumption that a gene is significantly overexpressed in an organ (statistical consideration), if its expression abundance accounts for the majority of that in all organs, say more than 70%, the gene is considered to be selectively expressed in that organ. The maximum FPKM value of a gene in any organs less than 5 was ignored because the expression level of the gene was supposed to be too low to analyze. Genes with FPKM above 5 were further analyzed. The means of a gene’s FPKM in all the organs were sorted. The organ with the median value and those with the biggest value were selected. Then, the expression level of the gene in the two organs (the highest and median organs) was compared with the Student t-test. The q-value, a false-discovery rate alternative to *p*-values, was also calculated as an adjustment for multiple comparisons [21]. If *p*-value and q-value were both less than 0.05, the gene was regarded as a candidate gene selectively expressed in the organ.

The means of the gene in all the organs were summed up as “Total”. The mean of the gene in the organ highest expressed it was regarded as “max mean”. Then, the MT ratio ((max mean)/total) was calculated. If the MT ratio was above 0.7, the gene was regarded as a selective gene in the organ. The gene’s product in the organ was regarded as an organic marker that may execute the selective function of the organ. The last reports on the relationship between the selective genes and the organs were searched at PubMed (www.pubmed.gov) on June 10, 2023.

The last report of the selective gene from the PubMed database was sought in the relative organ by searching the gene name and the organ both in the fields of title or abstract.

### 2.5 KEGG, and GO Analysis

The values of a gene in all the organs were sorted by its mean, and the organ that expressed the median value and that expressed the biggest value were selected. The expression abundance of the gene in the two organs was compared with the Student t-test. If there was significance (*p* < 0.05), the gene in the organ was regarded as an interesting gene. Interesting genes expressed in an organ were further analyzed to enrich the selective Kyoto Encyclopedia of Genes and Genomes (KEGG, https://www.kegg.jp/) and Gene Ontology (GO, http://www.geneontology.org/) pathways. KEGG enrichment [22] and KOG enrichment [23, 24] were performed by ClusterProfiler [25]. GO [26, 27] enrichment was performed by TopGO. The *p*-value and q-value were also calculated using the software mentioned above.

## 3 RESULTS

### 3.1 Total FPKM Distribution

**Fig. (1):**
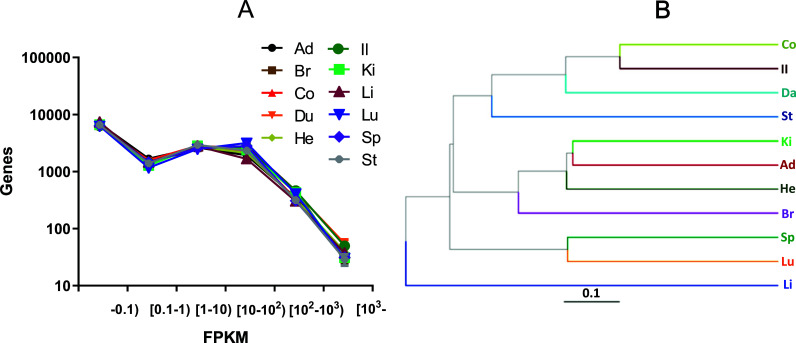
Distribution of gene expression and clustering analysis was made from 32,623 genes’ transcripts detected. The distribution of gene expression in different organs was similar (Mean ± SD, n = 3) (**A**). However, the function of the organs was different based on the clustering analysis of total gene expression from 11 organs (n = 3) (**B**). **Abbreviations:** Ad, adrenal gland; Br, brain; Co, colon; Du, duodenum; He, heart; Il, ileum; Ki, kidney; Li, liver; Lu, lung; Sp, spleen; St, stomach.

In the normal rats, 32,623 genes’ transcripts were detected, and most genes were expressed at a very low level (FPKM < 1), only a small portion of genes expressed at a very high level (FPKM > 1000) (Fig. **1A**). The overall FPKM distribution of every organ was similar. However, organs’ function is believed to be different, which suggests that the gene most highly expressed in one organ could be different from that in the other. According to the results of cluster analysis at the expression level (Fig. **1B**), the function of the colon is near the ileum, then to the duodenum and stomach, which is easy to be understood. The function of the kidney is near to the adrenal gland, then to the heart and brain; and the spleen's function is near to the lung. To our surprise, the function of the liver was far from that of the other organs.

### 3.2 Genes with Description Selectively Expressed in Different Organs

**Fig. (2):**
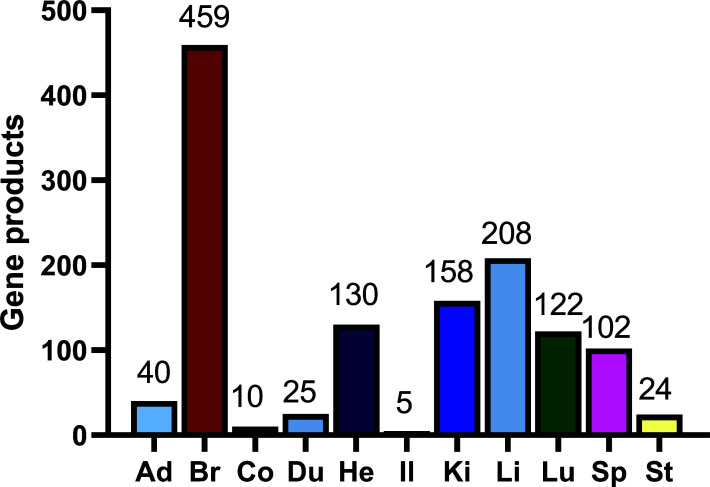
Genes selectively expressed in different organs based on their abundance. **Abbreviations:** Ad, adrenal gland; Br, brain; Co, colon; Du, duodenum; He, heart; Il, ileum; Ki, kidney; Li, liver; Lu, lung; Sp, spleen; St, stomach.

There were 15,922 genes with FPKM in any organ above 5, and 14,115 genes were significantly (*p* < 0.05) highly expressed in an organ. Among them, there were 12,617 genes accepted with q < 0.05. Apart from 123 genes without description, there were 1,283 genes with description selectively expressed in 11 organs (Fig. **2**). From the results from Fig. (**2**), the brain (Br) was the organ with the most complex function because 459 genes were selectively expressed in it. Instead, the gastrointestinal tracts, including the stomach (St), duodenum (Du), ileum (Il), and colon (Co), selectively expressed fewer genes, suggesting that their functions could be relatively simple or similar to other organs.

The total genes selectively expressed or the top 20 (if more) in 11 organs are listed in Tables **1**-**11**. Their full lists can be seen in the supplementary data. According to the description of the gene name, most selective genes were associated with the known specific functions of the organ. For example, Mgarp (mitochondria-localized glutamic acid-rich protein) in the adrenal gland (Table **1**) is associated with steroidogenesis [28]; Scg3 (secretogranin III) in the brain (Table **2**) with neuroendocrine [29]; Reg3g (regenerating islet-derived 3 gamma) in the colon (Table **3**) with intestinal bacterial translocation to the mesenteric lymph nodes [30]; Gip (gastric inhibitory polypeptide) in the duodenum (Table **4**) with regulation of insulin secretion [31]; Klhl38 (kelch-like family member 38) in the heart (Table **5**), though seldom reported, could be associated with the reversion of striated muscle atrophy [32]; Defa24 (defensin alpha 24) in the ileum (Table **6**) with intestinal barrier [33]; Slc3a1 [solute carrier family 3 (amino acid transporter heavy chain), member 1] in the kidney (Table **7**) with the transport of cystine and other amino acids across the membrane [34]; C5 (hemolytic complement) in the liver (Table **8**) was early verified to execute innate immune [35]; Icam1 (intercellular adhesion molecule 1) in the lung (Table **9**) with innate immune [36]; Coch (cochlin) used to highly expressed in the inner ear [37] also highly expressed in the spleen (Table **10**); and Cxcl17 (chemokine (C-X-C motif) ligand 17) in the stomach (Table **11**) with its innate immune [38]. Nevertheless, there were many genes that were not reported in the relative organs (supplementary data).

### 3.3 KEGG and GO Pathway Enrichment

#### 3.3.1 KEGG Pathway Enrichment

KEGG is a bioinformatics database resource for understanding high-level functions and utilities of the biological system, which includes the cell, the organism, and the ecosystem, from molecular-level information, especially large-scale molecular datasets generated by genome sequencing and other high-throughput experimental technologies. The selective KEGG pathways were enriched based on the abundance of genes most highly expressed in organs. The number of the selective pathway is listed in Fig. (**3**) and the top 20 pathways are listed in Tables **12**-**22**. Their full lists can be seen in the supplementary data. There were 179 “selective” pathways in 11 rat organs. Among them, 52 pathways were involved in two organs, 7 in three organs, and 1 in four organs. It should be noted that the “selective” pathways engaged in two or more organs were based on enrichment analysis. As can be seen from Fig. (**3**), organs with many selective pathways, like the brain, indicate that they undertake many complex functions. Conversely, organs with few selective pathways, like the adrenal glands and stomach, indicate their relatively simple functions. The results in Fig. (**3**), suggested that the lung could be the top 2 organs with the complex functions of the 11 organs.

The function of some pathways was verified in relative organs based on common understandings, for example, ko04925 (Aldosterone synthesis and secretion) in the adrenal gland (Table **12**), ko04721 (Synaptic vesicle cycle) in the brain (Table **13**), ko04672 (Intestinal immune network for IgA production) in the colon (Table **14**), ko04975 (Fat digestion and absorption) in the duodenum (Table **15**), ko04260 (Cardiac muscle contraction) in the heart (Table **16**), ko00520 (Amino sugar and nucleotide sugar metabolism) in the ileum (Table **17**), ko04964 (Proximal tubule bicarbonate reclamation) in the kidney (Table **18**), ko04976 (Bile secretion) in the liver (Table **19**), ko04151 (PI3K-Akt signaling pathway) in the lung (Table **20**), ko04640 (Hematopoietic cell lineage) in the spleen (Table **21**), and ko04971 (Gastric acid secretion) in the stomach (Table **22**).

#### 3.3.2 GO Pathway Enrichment

The GO database is the world’s largest source of bio-information on the functions of genes. This knowledge of the genes is a foundation for computational analysis of large-scale molecular biology and genetics experiments in biomedical research. Selective GO pathways were enriched based on the abundance of genes most highly expressed in organs. The number of the selective pathway is listed in Fig. (**4**) and the pathways of the adrenal gland, brain, colon, duodenum, heart, ileum, kidney, liver, lung, spleen, and stomach are listed in Tables **23**-**33**, respectively. There were 4,432 relatively selective pathways in 11 rat organs. Among them, 971 pathways were involved in two organs, 357 in three organs, 86 in four organs, 21 in five organs, 7 in six organs, and 1 in seven organs. It should be noted that the “selective” pathways are involved in two or more organs based on the enrichment analysis.

As can be seen from Fig. (**4**), organs with many selective pathways, like the lung, spleen and brain, indicate that they undertake many complex functions. Conversely, organs with few selective pathways, like the stomach and adrenal glands, indicate their relative sample functions. The results in Fig. (**3**), is similar to those in Fig. (**4**).

The top 20 GO pathways are shown in Tables **23**-**33**, and their full lists can be seen in the supplementary data. As for the top 20 GO pathways, the adrenal gland (Table **23**), colon (Table **25**), and kidney (Table **29**) had no real selective pathways, and the brain had the most selective pathways, suggesting that the brain has specific functions (Table **24**). According to the results of GO enrichment, the adrenal gland is a hypermetabolic organ because mitochondria in the organ are very active (Table **23**); the brain is a neural organ (Table **24**), which is well-accepted by scientists; the colon is an immune and metabolic organ (Table **25**); the duodenum is mainly an immune organ (Table **26**); the heart is also a hypermetabolic organ (Table **27**); the ileum is primarily an organ associated with protein synthesis, immune, and digestion (Table **28**); the kidney (Table **29**) and liver (Table **30**) are mainly an organ associated with metabolism; the lung is an organ mainly associated with angiogenesis and blood circulation (Table **31**); the spleen is an organ mainly associated with organelle metabolism (Table **32**), and the stomach is an organ mainly associated with digestion and glandular secretion (Table **33**).

### 3.4 Genes without Description but Selectively Expressed

**Fig. (5):**
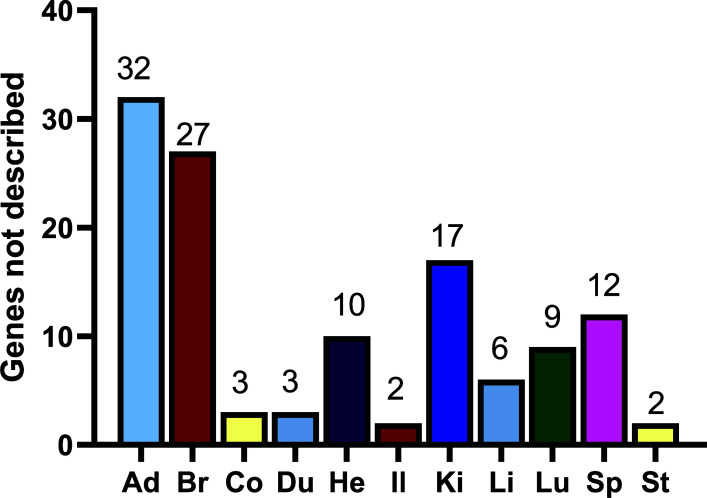
There were 123 Genes without description but selectively expressed in different organs based on their abundance. **Abbreviations:** Ad, adrenal gland; Br, brain; Co, colon; Du, duodenum; He, heart; Il, ileum; Ki, kidney; Li, liver; Lu, lung; Sp, spleen; St, stomach.

Apart from the genes whose function is described, there were 123 genes without a clear description but selectively expressed in 11 organs (Fig. **5**). From the results of Fig. (**5**), most genes without description were selectively expressed in the adrenal gland and brain. Instead, there were fewer genes without description in rat gastrointestinal tracts, including stomach, duodenum, ileum, and colon. The top 20 genes without description in the adrenal gland, brain, colon, duodenum, heart, ileum, kidney, liver, lung, spleen, and stomach were listed in Tables **34**-**44**, respectively; and their full lists can be seen in the supplementary data. Because the genes were not described but selectively expressed in the organs, their products and functions need further investigation. Given the low number of genes selectively expressed in the adrenal gland, the high number of undescribed high expression of genes in this organ suggests that the organ may be less studied.

## 4 DISCUSSION

Screening selectively expressed genes in organs is not only a tough task but also meaningful work because the results of the work will provide useful clues and even evidence for scientists to unveil the mechanism behind the overall dysfunction and symptoms. At least, we can obtain the putative organic markers for evaluating organic injury. There were good examples of some proteins selectively expressed in organs that were used as disease markers [8, 10-12] or used as therapeutic targets like trastuzumab on HER2 to treat breast cancer [167]. However, many selective genes have still not been revealed.

The present study screened out 1,406 genes selectively expressed in 11 rat organs, among which, 1,283 genes’ function was described, and 123 of which still need to be described in the near future. Some of the genes’ function was confirmed in the organs that were noted in Tables **1**-**11**, but a good portion of them or the relationship between their function and the organs was not addressed. The new findings are useful to unveil the mechanism of their organic functions. Unfortunately, as for the selective genes in organs mentioned in the introduction, only troponin [10] was proved to be selective by the present study, and NeuN in the brain [8], GPT in the liver [11], and NGAL in the kidney [12] were not included in the present list of the selective genes. After consulting the FPKM values, it is exactly that the FPKM of NeuN in the brain was the highest, but not significant. The relative neuronal marker was further proved by recent work [9]. The highest GPT (GPT2) in the liver was significant, but the level of expression was not dominant (only about 45% of the total). Of course, if the criterion of selective genes was lowered, more genes would be included in the selective gene list, namely, in the list of putative organic markers. Phosphodiesterase 5 (PDE5a), an enzyme associated with angiectasis, is another similar example. PDE5a was verified to be the most highly expressed gene in the lung, but not included in the selective gene list (Table **9**), supporting PDE5 inhibitors’ pharmacological effect on pulmonary arterial hypertension [168, 169].

The selective genes and their products can be used as physiological or disease markers. If a cell is injured, the selective gene’s product normally existing in its cytoplasm will be released to the blood. Based on the principle, some injury markers like serum Myl3 protein for heart injury [170] were screened out and verified by the present study. Theoretically, products from selective genes can be used as disease markers. However, it should be noted that because of some genes expressed in rats (*e.g*., Uox in the liver) [171], but not in humans, the fact that the products from the selective genes used as disease markers are only advisory, needing further verification.

The functional pathways of an organ enriched by the highest-expressed genes were largely supported by the known understanding. However, there are still some interesting functions that were not focused on. For example, KEGG pathways (Tables **12**-**22**) like ko00061 (fatty acid biosynthesis) in the adrenal gland, ko04911 (insulin secretion) in the brain, ko00280 (Valine, leucine, and isoleucine degradation) in the heart and kidney, and ko04360 (axon guidance) in the lung were seldom paid attention to by scientists. Similar results would be obtained in the results of GO pathways (Table **23**-**33**). The unpopular organic functional pathways enriched by the present study would open a new window to make insight into their mechanism. Especially the adrenal glands may be an organ with few basic researches.

Though the selective genes and the interesting genes only existed in one organ, the organic pathways including KEGG (Tables **12**-**22**) and GO (Tables **23**-**33**) pathways, enriched by them could exist in two or more organs. Since a pathway often involves many proteins, it is theoretically different for the real functions of the same selective pathway enriched by different selective genes. The same pathway is enriched in different organs with different profiles. Anyway, the functions are different from organ to organ, although they share some similarities at pathway levels.

## CONCLUSION

In the end, because there were no standard criteria ready to evaluate a gene's selectivity, the present study used the dominant portion of FPKM value and statistical analysis. If the FPKM value of a gene in an organ accounted for 70% of the total values of all the organs concerned, the gene was assumed as the selective gene in the organ after excluding genes with low abundance. If the criterion were lowered, the list of the selective genes would be lengthened. On the other hand, the selective genes screened out by the present study were only based on the results of 11 organs in male rats, and some selective genes in other organs or female rats were neglected or missed. Moreover, the weights of the organs were not taken into account in the present study. Considering that the genome of rats has approximately 85% similarity with that of humans, this study provides a useful exploration of human organic markers and organ function, though the selective genes, the putative markers, and the functional pathways suggested are only advisory and worthy of further investigation.

## Figures and Tables

**Fig. (3):**
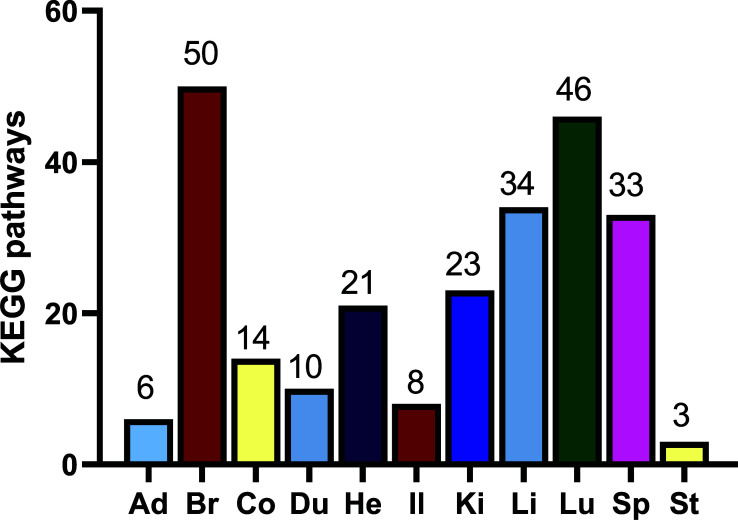
Selective KEGG enrichment in different organs was based on the abundance of genes most highly expressed in organs. **Abbreviations:** Ad, adrenal gland; Br, brain; Co, colon; Du, duodenum; He, heart; Il, ileum; Ki, kidney; Li, liver; Lu, lung; Sp, spleen; St, stomach.

**Fig. (4):**
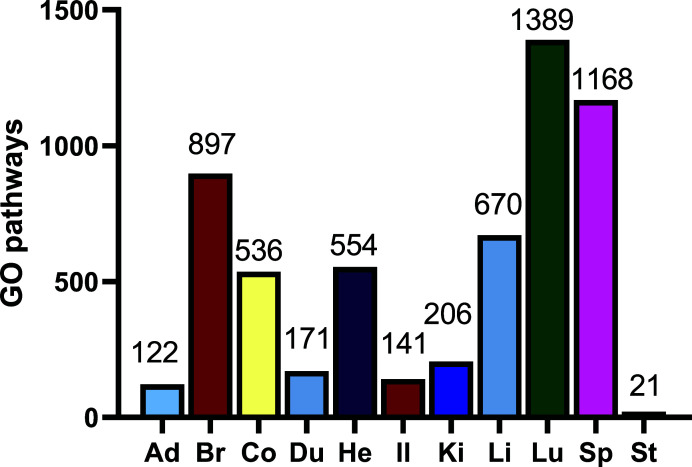
Selective GO enrichment in different organs based on the abundance of genes most highly expressed in organs. **Abbreviations:** Ad, adrenal gland; Br, brain; Co, colon; Du, duodenum; He, heart; Il, ileum; Ki, kidney; Li, liver; Lu, lung; Sp, spleen; St, stomach.

**Table 1:** Top 20 of 40 genes with description selectively expressed in the adrenal gland (Ad) based on their abundance (n = 3).

**No.**	**Gene Name**	**Product (Description)**	Last Ref.*	**Median Organ**	**FPKM**		***p*-value**	**q-value**	**Mean/total**
					**Mean**	**Total**	-	-	-
1	Mgarp	Mitochondria-localized glutamic acid-rich protein	[39]	He	469.5	473.5	1.018E-06	0.000	0.992
2	Lrcol1	Leucine rich colipase-like 1	-	Ki	7.6	7.8	1.165E-06	0.000	0.967
3	Cyp21a1	Cytochrome P450, family 21, subfamily a, polypeptide 1	[40]	St	9139.9	9148.9	2.277E-06	0.001	0.999
4	Akr1b7	Aldo-keto reductase family 1, member B7	[41]	Ki	2280.6	2281.5	5.124E-06	0.001	1.000
5	Cyp11b2	Cytochrome P450, family 11, subfamily b, polypeptide 2	[42]	Lu	327.8	337.2	5.121E-05	0.003	0.972
6	Mir450a1	MicroRNA 450a1	-	St	12.8	13.2	9.565E-05	0.004	0.967
7	Star	Steroidogenic acute regulatory protein	[43]	St	1438.9	1457.2	1.444E-04	0.005	0.987
8	Ceacam16	Carcinoembryonic antigen-related cell adhesion molecule 16	-	Co	73.6	74.6	1.595E-04	0.005	0.986
9	Mrap	Melanocortin 2 receptor accessory protein	[44]	St	413.8	448.9	1.809E-04	0.006	0.922
10	Nkain3	Na+/K+ transporting ATPase interacting 3	-	St	6.6	7.9	2.328E-04	0.006	0.837
11	Nr0b1	Nuclear receptor subfamily 0, group B, member 1	[45]	Co	41.1	42.0	2.828E-04	0.007	0.979
12	Pbx4	Pre-B-cell leukemia homeobox 4	-	Du	15.4	21.5	3.187E-04	0.007	0.715
13	Slc27a3	Solute carrier family 27 (fatty acid transporter), member 3	-	St	141.2	167.2	3.292E-04	0.007	0.844
14	Mc2r	Melanocortin 2 receptor (adrenocorticotropic hormone)	[46]	St	58.6	63.3	3.388E-04	0.008	0.925
15	Eepd1	Endonuclease/exonuclease/phosphatase family domain containing 1	-	Co	561.4	668.3	3.895E-04	0.008	0.840
16	Nr5a1	Nuclear receptor subfamily 5, group A, member 1	[47]	Br	51.8	61.4	3.918E-04	0.008	0.843
17	Tmem200a	Transmembrane protein 200A	-	St	23.9	30.3	4.488E-04	0.009	0.789
18	LOC108348086	Hydroxy-delta-5-steroid dehydrogenase, 3 beta- and steroid delta-isomerase 2	-	Du	557.5	558.7	4.900E-04	0.009	0.998
19	Fdx1	Ferredoxin 1	[48]	Du	2301.0	2657.4	5.368E-04	0.010	0.866
20	Cyp11a1	Cytochrome P450, family 11, subfamily a, polypeptide 1	[49]	Co	4795.7	4802.2	5.905E-04	0.010	0.999

**Note:** Sorted by q-value. Br, brain; Co, colon; Du, duodenum; He, heart; Ki, kidney; Lu, lung; St, stomach.

* Last Ref. was based on the reports documented in PubMed (www.pubmed.gov) before June 10, 2023.

**Table 2:** Top 20 of 459 genes with description selectively expressed in the brain (Br) based on their abundance (n = 3).

**No.**	**Gene Name**	**Product (Description)**	Last Ref.*	**Median Organ**	**FPKM**		***p*-value**	**q-value**	**Mean/total**
					**Mean**	**Total**			
1	Dio2	Preoptic regulatory factor 1	[50]	Sp	11.5	14.1	5.821E-08	3.324E-07	0.82
2	Scg3	Secretogranin III	[51]	Du	197.4	215.7	4.057E-09	1.592E-05	0.91
3	Gabbr1	Gamma-aminobutyric acid (GABA) B receptor 1	[52]	Ad	448.3	627.1	2.797E-06	2.563E-05	0.71
4	Asic2	Acid-sensing (proton-gated) ion channel 2	[53]	Co	19.1	25.8	8.626E-08	9.567E-05	0.74
5	Adcyap1r1	Adenylate cyclase-activating polypeptide 1 receptor type 1	[54]	Co	36.9	44.8	1.593E-07	1.165E-04	0.82
6	Chst10	Carbohydrate sulfotransferase 10	[55]	He	37.1	47.6	3.197E-06	3.522E-04	0.78
7	Larp6	La ribonucleoprotein domain family, member 6	-	St	17.8	24.1	3.871E-06	3.944E-04	0.74
8	Vsnl1	Visinin-like 1	[56]	Il	406.5	450.9	1.252E-06	4.572E-04	0.90
9	Snap91	Synaptosomal-associated protein 91	[57]	Co	139.3	148.7	1.341E-06	4.692E-04	0.94
10	Tceal3	Transcription elongation factor A (SII)-like 6	[58]	He	132.8	141.2	2.179E-06	5.977E-04	0.94
11	Pdzd4	PDZ domain containing 4	[59]	Du	42.5	50.0	2.611E-06	6.401E-04	0.85
12	LOC100911402	Cell cycle exit and neuronal differentiation 1	-	He	231.8	236.2	3.063E-06	6.991E-04	0.98
13	Acsbg1	Acyl-CoA synthetase bubblegum family member 1	-	Lu	106.6	126.1	3.093E-06	7.091E-04	0.85
14	Gdap1l1	Ganglioside-induced differentiation-associated protein 1-like 1	[60]	Du	70.6	77.1	3.576E-06	7.453E-04	0.92
15	Adgrb3	Adhesion G protein-coupled receptor B3	[61]	Du	22.1	22.8	3.932E-06	7.981E-04	0.97
16	Fam131b	Family with sequence similarity 131, member B	[62]	Lu	56.2	58.1	3.942E-06	8.091E-04	0.97
17	Plp1	Proteolipid protein 1	[63]	He	1572.6	1599.8	4.805E-06	8.959E-04	0.98
18	Nipal4	NIPA-like domain containing 4	-	Du	6.3	7.9	1.199E-05	9.210E-04	0.79
19	RragB	Ras-related GTP-binding protein B-like	[64]	He	15.2	19.4	2.190E-05	1.143E-03	0.78
20	Stmn3	Stathmin-like 3	[65]	Du	887.3	918.2	8.338E-06	1.180E-03	0.97

**Note:** Sorted by q-value. Ad, adrenal gland; Co, colon; Du, duodenum; He, heart; Il, ileum; Ki, kidney; Lu, lung; Sp, spleen; St, stomach.

* Last Ref. was based on the reports documented in PubMed (www.pubmed.gov) before June 10, 2023.

**Table 3:** Top genes with description selectively expressed in the colon (Co) based on their abundance (n = 3).

**No**	**Gene Name**	**Product (Description)**	Last Ref.*	**Median Organ**	**FPKM**		***p*-value**	**Q-value**	**Mean/total**
					**Mean**	**Total**			
1	Reg3g	Regenerating islet-derived 3 gamma	[66]	Lu	9161.7	12157.8	1.46E-08	4.93E-05	0.754
2	Reg3b	Regenerating islet-derived 3 beta	[66]	Br	6569.9	8784.4	6.70E-06	1.06E-03	0.748
3	St6galnac1	ST6 (alpha-N-acetyl-neuraminyl-2,3-beta-galactosyl-1,3)-N- acetylgalactosaminide alpha-2,6-sialyltransferase 1	[67]	Br	286.1	294.8	7.56E-06	1.12E-03	0.971
4	Ighg	Immunoglobulin heavy chain (gamma polypeptide)	[68]	Br	78.0	106.8	7.38E-05	2.67E-03	0.730
5	Hmcn2	Hemicentin 2	-	St	30.5	31.2	1.55E-04	5.12E-03	0.977
6	LOC290595	Hypothetical gene supported by AF152002	-	Ad	103.0	146.0	1.75E-04	5.46E-03	0.706
7	Ace	Angiotensin I converting enzyme	[69]	St	51.5	59.7	6.53E-04	1.06E-02	0.861
8	LOC691670	Similar to natural killer cell protease 7	-	Sp	11.1	15.4	6.77E-03	3.56E-02	0.724
9	Fgf19	Fibroblast growth factor 19	[70]	Ad	41.2	43.2	9.63E-03	4.32E-02	0.953
10	Mir192	MicroRNA 192	[71]	Ad	6.9	6.9	1.17E-02	4.80E-02	1.000

**Note:** Sorted by q-value. Ad, adrenal gland; Br, brain; Lu, lung; Sp, spleen; St, stomach.

* Last Ref. was based on the reports documented in PubMed (www.pubmed.gov) before June 10, 2023.

**Table 4:** Top 20 of 25 genes with description selectively expressed in the duodenum (Du) based on their abundance (n = 3).

**No.**	**Gene Name**	**Product (Description)**	Last Refs.*	**Median Organ**	**FPKM**		***p*-value**	**q-value**	**Mean/total**
					**Mean**	**Total**			
1	Gip	Gastric inhibitory polypeptide	[72]	Ad	79.2	81.4	7.42E-06	1.11E-03	0.973
2	LOC100910259	Liver carboxylesterase-like	-	Sp	498.7	699.5	5.32E-05	2.99E-03	0.713
3	Prap1	Proline-rich acidic protein 1	-	Br	4433.8	4622.2	9.65E-05	4.04E-03	0.959
4	Papss2	3'-phosphoadenosine 5'-phosphosulfate synthase 2	-	Sp	649.9	779.5	1.20E-04	4.51E-03	0.834
5	Tm4sf5	Transmembrane 4 L six family member 5	-	Ad	940.9	1272.0	1.36E-04	4.81E-03	0.740
6	RGD1311933	Similar to RIKEN cDNA 2310057J18	-	Ad	221.2	221.9	2.62E-04	6.69E-03	0.997
7	Cyp2c7	Cytochrome P450, family 2, subfamily c, polypeptide 7	-	Ad	48.0	50.7	3.56E-04	7.82E-03	0.947
8	Aadac	Arylacetamide deacetylase	-	St	96.4	133.8	6.93E-04	1.10E-02	0.720
9	Tmprss15	Transmembrane protease, serine 15	-	Sp	138.8	139.2	7.87E-04	1.17E-02	0.997
10	RGD1561551	Similar to Hypothetical protein MGC75664	-	Ad	842.1	842.9	1.28E-03	1.50E-02	0.999
11	Alppl2	Alkaline phosphatase, placental-like 2	-	Co	60.3	71.1	1.40E-03	1.57E-02	0.848
12	Akp3	Alkaline phosphatase 3, intestine, not Mn requiring	[73]	Ad	2279.3	2280.1	1.67E-03	1.72E-02	1.000
13	Ada	Adenosine deaminase	[74]	Ki	1461.9	2071.3	1.74E-03	1.76E-02	0.706
14	Bco1	Beta-carotene oxygenase 1	[75]	Ki	160.1	210.3	1.78E-03	1.78E-02	0.761
15	Slc4a7	Solute carrier family 4, sodium bicarbonate cotransporter, member 7	[75, 76]	St	108.5	137.6	1.90E-03	1.79E-02	0.789
16	Alpi	Alkaline phosphatase, intestinal	[77]	Br	1098.5	1193.8	1.84E-03	1.81E-02	0.920
17	Treh	Trehalase (brush-border membrane glycoprotein)	[78]	Ki	260.7	268.5	2.44E-03	2.09E-02	0.971
18	Trpv6	Transient receptor potential cation channel, subfamily V, member 6	[79]	Sp	24.2	32.7	2.45E-03	2.10E-02	0.741
19	Otop3	Otopetrin 3	-	Co	69.3	70.2	3.51E-03	2.53E-02	0.987
20	Pdx1	Pancreatic and duodenal homeobox 1	[80]	Ad	58.6	61.7	4.91E-03	3.02E-02	0.950

**Note:** Sorted by q-value. Ad, adrenal gland; Br, brain; Co, colon; Ki, kidney; Sp, spleen; St, stomach.

* Last Refs. was based on the reports documented in PubMed (www.pubmed.gov) before June 10, 2023.

**Table 5:** The top 20 of 130 genes with description are selectively expressed in the heart (He) based on their abundance(n = 3).

**No.**	**Gene Name**	**Product (Description)**	Last Ref.*	**Median Organ**	**FPKM**		***p*-value**	**q-value**	**Mean/total**
					**Mean**	**Total**			
1	Klhl38	Kelch-like family member 38	[81]	St	10.4	13.4	4.25E-07	1.64E-04	0.776
2	Rbm24	RNA binding motif protein 24	[82, 83]	Co	45.0	58.7	7.37E-07	1.98E-04	0.768
3	Ldb3	LIM domain binding 3	[84]	St	541.1	590.9	5.15E-07	2.81E-04	0.916
4	LOC100909784	Leiomodin 2 (cardiac)	-	St	92.6	93.6	5.36E-07	2.99E-04	0.989
5	Hspb2	Heat shock protein B2	[85]	St	183.4	212.0	2.62E-06	4.35E-04	0.865
6	Itgb1bp2	Integrin beta 1 binding protein 2	[86]	Du	121.2	140.6	1.30E-06	4.44E-04	0.862
7	Klhl31	Kelch-like family member 31	[87]	Sp	57.0	58.4	1.22E-06	4.49E-04	0.975
8	Tnni3k	TNNI3 interacting kinase	[88]	Ad	85.4	86.6	1.26E-06	4.58E-04	0.986
9	Pla2g5	Phospholipase A2, Group V	[89]	Sp	54.0	60.0	2.19E-06	4.89E-04	0.899
10	Fsd2	Fibronectin type III and SPRY domain containing 2	[90]	Du	47.6	48.3	1.87E-06	5.56E-04	0.986
11	Tmem182	Transmembrane protein 182	[91]	Ki	79.9	84.0	2.18E-06	5.88E-04	0.951
12	Rd3l	Retinal degeneration 3-like	-	Ad	18.5	23.6	7.90E-06	1.10E-03	0.785
13	Nkx2-5	NK2 homeobox 5	[92]	Lu	75.9	84.4	7.41E-06	1.11E-03	0.899
14	Sgcg	Sarcoglycan, gamma	[93]	Il	93.4	110.5	1.83E-05	1.13E-03	0.845
15	Hhatl	Hedgehog acyltransferase-like	[94]	Ki	119.2	133.1	8.86E-06	1.22E-03	0.896
16	Cav3	Caveolin 3	[95]	Sp	116.1	123.7	9.50E-06	1.24E-03	0.939
17	LOC691485	Hypothetical protein LOC691485	-	Br	24.1	29.9	2.37E-05	1.26E-03	0.807
18	Kbtbd12	Kelch repeat and BTB (POZ) domain containing 12	-	St	16.2	18.2	1.23E-05	1.35E-03	0.891
19	Txlnb	Taxilin beta	-	Co	68.5	69.3	1.57E-05	1.62E-03	0.987
20	Spink8	Serine peptidase inhibitor, Kazal type 8	-	Br	166.1	187.3	2.10E-05	1.74E-03	0.887

**Note:** Sorted by q-value. Ad, adrenal gland; Br, brain; Co, colon; Du, duodenum; Il, ileum; Ki, kidney; Lu, lung; Sp, spleen; St, stomach.

* Last Ref. was based on the reports documented in PubMed (www.pubmed.gov) before June 10, 20123.

**Table 6:** Top genes with description selectively expressed in the ileum (Il) based on their abundance (n = 3).

**No.**	**Gene Name**	**Product (Description)**	Last Ref.*	**Median Organ**	**FPKM**		***p*-value**	**q-value**	**Mean/total**
					**Mean**	**Total**			
1	LOC100910656	rCG60069-like	-	Sp	244.0	341.0	0.001	0.011	0.715
2	Defa24	Defensin alpha 24	-	Ad	15591.7	19391.4	0.001	0.011	0.804
3	Fabp6	Fatty acid binding protein 6, ileal	[96]	Ki	51493.4	56686.5	0.001	0.012	0.908
4	Defal1	Defensin alpha-like 1	-	Ad	29241.0	34877.4	0.001	0.015	0.838
5	Pla2g4c	Phospholipase A2, group IVC-like 1	-	St	26.1	30.0	0.005	0.030	0.869

**Note:** Sorted by q-value. Ad, adrenal gland; Ki, kidney; Sp, spleen; St, stomach.

* Last Ref. was based on the reports documented in PubMed (www.pubmed.gov) before June 10, 2023.

**Table 7:** Top 20 of 158 genes with description selectively expressed in the kidney (Ki) based on their abundance(n = 3).

**No.**	**Gene Name**	**Product (Description)**	Last Ref.*	**Median Organ**	**FPKM**		***p*-value**	**q-value**	**Mean/total**
					**Mean**	**Total**			
1	C1qtnf3	C1q and tumor necrosis factor-related protein 3	-	He	32.5	42.7	7.02E-07	7.13E-07	0.760
2	Pter	Phosphotriesterase related	[97]	Co	184.9	239.7	1.00E-08	2.91E-06	0.771
3	Gclc	Glutamate-cysteine ligase, catalytic subunit	[98]	Ad	1920.8	2266.6	1.91E-08	1.55E-05	0.847
4	Slc3a1	Solute carrier family 3 (amino acid transporter heavy chain), member 1	[99]	He	1569.4	1923.0	5.63E-09	3.01E-05	0.816
5	Trpv4	Transient receptor potential cation channel, subfamily V, member 4	[100]	St	30.2	37.6	1.65E-06	4.90E-05	0.803
6	Skint10	Selection and upkeep of intraepithelial T cells 10	-	Ad	5.3	5.5	2.71E-08	6.73E-05	0.965
7	LOC688553	Hypothetical protein LOC688553	-	Du	62.3	71.8	1.07E-06	1.09E-04	0.868
8	Stra6	Stimulated by retinoic acid 6	[101]	Sp	22.4	25.8	4.59E-07	1.45E-04	0.868
9	RGD1310495	Similar to KIAA1919 protein	-	Il	71.5	82.3	1.64E-07	1.64E-04	0.869
10	Wdr72	WD repeat domain 72	[102]	Il	8.0	9.9	1.94E-07	1.80E-04	0.805
11	Haao	3-hydroxyanthranilate 3,4-dioxygenase	[103]	Il	444.3	616.7	2.08E-07	1.86E-04	0.720
12	Emx2	Empty spiracles homeobox 2	[104]	St	13.9	16.2	2.79E-07	2.16E-04	0.857
13	Gba3	Glucosidase, beta, acid 3	[105]	Ad	172.9	173.3	3.61E-07	2.45E-04	0.998
14	Car12	Carbonic anyhydrase 12	[106]	Ad	352.9	454.9	2.45E-06	2.67E-04	0.776
15	Pdzk1ip1	PDZK1 interacting protein 1	[107]	Du	390.1	434.3	4.90E-07	2.77E-04	0.898
16	Spo11	SPO11 meiotic protein covalently bound to DSB	[108]	Br	6.9	8.8	2.97E-06	2.93E-04	0.787
17	Slc6a18	Solute carrier family 6 (neutral amino acid transporter), member 18	[109]	Ad	273.3	274.3	7.07E-07	3.44E-04	0.996
18	Glyat	Glycine-N-acyltransferase	[110]	Ad	756.4	947.6	1.17E-06	4.42E-04	0.798
19	Aspa	Aspartoacylase	[111]	Li	140.1	199.1	4.18E-06	5.53E-04	0.703
20	Cyp4a2	Cytochrome P450, family 4, subfamily a, polypeptide 2	[112]	Co	561.2	737.0	2.15E-06	5.99E-04	0.761

**Note:** Sorted by q-value. Ad, adrenal gland; Br, brain; Co, colon; Du, duodenum; He, heart; Il, ileum; Li, liver; Sp, spleen; St, stomach.

* Last Ref. was based on the reports documented in PubMed (www.pubmed.gov) before June 10, 2023.

**Table 8:** Top 20 of 208 genes with description selectively expressed in the liver (Li) based on their abundance (n = 3).

**No**	**Gene Name**	**Product (Description)**	Last Ref.*	**Median Organ**	**FPKM**		***p*-value**	**q-value**	**Mean/total**
					**Mean**	**Total**			
1	C5	Hemolytic complement	[113]	Sp	118.2	142.0	9.584E-10	1.152E-05	0.833
2	Serpind1	Serpin peptidase inhibitor, clade D (heparin cofactor), member 1	[114]	Ad	389.6	390.6	9.631E-08	1.267E-04	0.997
3	Saa4	Hermansky-Pudlak syndrome 5	[115]	Ki	691.8	743.6	1.417E-07	1.510E-04	0.930
4	Crp	C-reactive protein, pentraxin-related	[116]	Ki	5777.4	5787.5	1.605E-07	1.636E-04	0.998
5	C8b	Complement component 8, beta polypeptide	[117]	Ad	295.8	297.0	1.661E-07	1.664E-04	0.996
6	C4bpa	Complement component 4 binding protein, alpha	[118]	He	295.7	308.0	2.467E-07	2.024E-04	0.960
7	Cfi	Complement factor I	[118]	Ki	469.3	534.4	4.264E-07	2.665E-04	0.878
8	C8g	Complement component 8, gamma polypeptide	[119]	Br	180.5	214.3	6.792E-07	3.024E-04	0.842
9	Slc13a4	Solute carrier family 13 (sodium/sulfate symporter), member 4	[120]	Il	27.9	38.9	6.273E-07	3.033E-04	0.718
10	Tmprss6	Transmembrane protease, serine 6	[121]	Il	170.2	171.0	5.620E-07	3.060E-04	0.995
11	Uroc1	Urocanate hydratase 1	-	Ki	100.5	101.0	6.137E-07	3.200E-04	0.995
12	Afm	Afamin	[122]	Br	694.3	744.9	6.160E-07	3.206E-04	0.932
13	Mug1	Alpha-1-inhibitor III	[123]	Ad	5659.4	5677.1	8.210E-07	3.702E-04	0.997
14	Mbl1	Mannose-binding lectin (protein A) 1	[124]	Sp	230.0	249.3	1.212E-06	4.497E-04	0.922
15	F10	Coagulation factor X	[125]	Il	292.5	297.8	1.706E-06	5.317E-04	0.982
16	LOC 100909524	Serpin peptidase inhibitor, clade A (alpha-1 antiproteinase, antitrypsin), member 10	-	Br	95.6	98.0	1.825E-06	5.477E-04	0.975
17	Slc38a4	Solute carrier family 38, member 4	[126]	St	209.4	212.6	1.996E-06	5.758E-04	0.985
18	Glyatl1	Glycine-N-acyltransferase-like 1	[127]	Il	109.9	116.8	2.111E-06	5.936E-04	0.941
19	C4bpb	Complement component 4 binding protein, beta	[118]	Ki	305.3	313.2	2.177E-06	6.020E-04	0.975
20	Pzp	Pregnancy-zone protein	[128]	Il	2009.8	2053.1	2.243E-06	6.122E-04	0.979

**Note:** Sorted by q-value. Ad, adrenal gland; Br, brain; He, heart; Il, ileum; Ki, kidney; Sp, spleen; St, stomach.

* Last Ref. was based on the reports documented in PubMed (www.pubmed.gov) before June 10, 2023.

**Table 9:** Top 20 of 122 genes with description selectively expressed in rat lung (Lu) based on their abundance (n = 3).

**No.**	**Gene Name**	**Product (Description)**	Last Ref.*	**Median Organ**	**FPKM**		***p*-value**	**q-value**	**Mean/total**
					**Mean**	**Total**			
1	St8sia2	ST8 alpha-N-acetyl-neuraminide alpha-2,8- sialyltransferase 2	[129]	Il	7.0	8.1	3.25E-07	6.25E-07	0.872
2	Ly6l	Lymphocyte antigen 6 family member L	[130]	Br	77.2	103.7	5.49E-08	5.61E-05	0.745
3	Icam1	Intercellular adhesion molecule 1	[131]	Il	242.5	329.3	1.12E-06	7.80E-05	0.736
4	LOC102546678	Proline-rich Gla (G-carboxyglutamic acid) 3 (transmembrane)	-	Il	18.0	20.5	1.97E-07	1.37E-04	0.879
5	LOC102554838	Stathmin domain-containing protein 1-like	-	Co	6.2	8.6	2.53E-07	2.02E-04	0.726
6	Thbd	Thrombomodulin	[132]	Il	297.8	387.8	1.70E-06	2.35E-04	0.768
7	Matn4	Matrilin 4	[133]	Ad	36.4	46.0	2.30E-06	3.47E-04	0.791
8	LOC681341	Similar to paired immunoglobin-like type 2 receptor β	-	Co	11.6	15.8	2.74E-06	3.79E-04	0.733
9	Prrg3	Proline-rich Gla (G-carboxyglutamic acid) 3 (transmembrane)	-	Co	17.5	19.4	1.66E-06	5.23E-04	0.903
10	Lhb	Luteinizing hormone beta polypeptide	[134]	Ki	10.3	13.9	9.59E-06	5.31E-04	0.746
11	Acvrl1	Activin A receptor type II-like 1	[135]	Ki	238.0	336.0	2.12E-06	5.85E-04	0.708
12	Pifo	Primary cilia formation	[136]	Ad	6.3	7.9	2.64E-06	6.65E-04	0.803
13	Scgb1a1	Secretoglobin, family 1A, member 1 (uteroglobin)	[137]	Ad	21465.0	21576.2	3.55E-06	7.70E-04	0.995
14	Fhad1	Forkhead-associated (FHA) phosphopeptide binding domain 1	-	Ki	9.2	10.7	5.42E-06	7.71E-04	0.854
15	Nme9	NME/NM23 family member 9	-	Ad	9.0	10.4	3.60E-06	7.76E-04	0.868
16	RGD1561648	RGD1561648	-	Co	7.6	10.6	9.11E-06	8.24E-04	0.718
17	LOC108348266	Cytochrome P450, family 2, subfamily b, polypeptide 1	-	Br	528.5	702.5	6.04E-06	1.00E-03	0.752
18	Dram1	DNA-damage regulated autophagy modulator 1	[138]	Ad	133.1	177.1	1.04E-05	1.04E-03	0.752
19	Limch1	LIM and calponin homology domains 1	[139]	St	174.7	216.9	8.99E-06	1.17E-03	0.805
20	LOC680885	Hypothetical protein LOC680885	-	Ad	14.2	15.3	1.09E-05	1.35E-03	0.928

**Note:** Sorted by q-value. Ad, adrenal gland; Br, brain; Co, colon; Il, ileum; Ki, kidney; St, stomach.

* Last Ref. was based on the reports documented in PubMed (www.pubmed.gov) before June 10, 2023.

**Table 10:** Top 20 of 102 genes with description selectively expressed in the spleen (Sp) based on their abundance (n = 3).

**No.**	**Gene Name**	**Product (Description)**	Last Ref.*	**Median Organ**	**FPKM**		***p*-value**	**q-value**	**Mean/total**
					**Mean**	**Total**			
1	Coch	Cochlin	[140]	Il	318.4	345.0	6.96E-11	6.89E-08	0.923
2	SNORD79	Small nucleolar RNA, C/D box 79	-	St	13.3	17.8	6.87E-06	1.07E-03	0.747
	Tlx1	T-cell leukemia, homeobox 1	[141]	Br	23.4	25.1	1.68E-05	1.66E-03	0.933
3	Erfe	Family with sequence similarity 132, member B	[142]	Du	11.6	13.0	3.33E-05	2.17E-03	0.892
4	Trim59	Tripartite motif-containing 59	[143]	Ki	104.2	131.3	4.80E-05	2.60E-03	0.794
5	Treml2	Triggering receptor expressed on myeloid cells-like 2	-	Du	27.9	35.3	6.25E-05	3.24E-03	0.790
	SNORA4	Small nucleolar RNA, H/ACA box 4	-	St	10.9	14.5	6.42E-05	3.29E-03	0.755
6	Spic	Spi-C transcription factor (Spi-1/PU.1 related)	[144]	Il	38.4	43.6	7.65E-05	3.57E-03	0.880
7	Adgre4	EGF-like module containing mucin-like, hormone receptor-like sequence 4	-	Du	26.0	34.9	1.44E-04	4.25E-03	0.743
8	Kel	Kell blood group, metallo-endopeptidase	[145]	Br	140.8	146.6	1.10E-04	4.31E-03	0.961
9	Tspo2	Translocator protein 2	-	Du	45.0	47.3	1.18E-04	4.44E-03	0.950
10	Defb36	Defensin beta 36	-	Ad	6.1	7.3	1.70E-04	5.38E-03	0.833
11	Icam4	Intercellular adhesion molecule 4, Landsteiner-Wiener blood group	[146]	Ad	20.1	22.7	1.93E-04	5.43E-03	0.884
12	Mylk2	Myosin light chain kinase 2	-	Ad	14.2	16.3	1.80E-04	5.49E-03	0.872
13	Epb42	Erythrocyte membrane protein band 4.2	-	Ki	88.5	91.6	1.95E-04	5.75E-03	0.966
14	Tnn	Tenascin N	[147]	Ad	8.0	9.3	2.22E-04	6.02E-03	0.862
15	Grap2	GRB2-related adaptor protein 2	-	Br	35.2	46.2	2.38E-04	6.32E-03	0.761
16	Cxcl6	Chemokine (C-X-C motif) ligand 6	[148]	St	6.3	8.0	3.06E-04	6.33E-03	0.791
17	Clec4m	CD209b antigen	[149]	Ki	45.6	46.6	2.35E-04	6.33E-03	0.978
18	LOC681325	Hypothetical protein LOC681325	-	He	17.2	20.7	2.59E-04	6.54E-03	0.830
19	Ahsp	Alpha hemoglobin stabilizing protein	[150]	St	2059.2	2118.7	2.61E-04	6.68E-03	0.972
20	Rhag	Rh-associated glycoprotein	[151]	Il	179.6	180.0	2.74E-04	6.84E-03	0.998

**Note:** Sorted by q-value. Ad, adrenal gland; Br, brain; Du, duodenum; He, heart; Il, ileum; Ki, kidney; St, stomach.

* Last Ref. was based on the reports documented in PubMed (www.pubmed.gov) before June 10, 2023.

**Table 11:** Top 20 of 24 genes with description selectively expressed in the stomach (St) based on their abundance (n = 3).

**No.**	**Gene Name**	**Product (Description)**	Last Ref.*	**Median Organ**	**FPKM**		***p*-value**	**q-value**	**Mean/total**
					**Mean**	**Total**			
1	Cxcl17	Chemokine (C-X-C motif) ligand 17	[152]	Br	822.1	1042.0	1.41E-09	1.07E-05	0.789
2	Kcnk16	Potassium channel, two pore domain subfamily K, member 16	-	Co	7.8	9.2	4.69E-07	1.11E-04	0.855
3	Anxa10	Annexin A10	[153]	Br	946.4	954.5	1.83E-06	5.52E-04	0.991
4	Fxyd3	FXYD domain-containing ion transport regulator 3	[154]	Li	1153.4	1435.8	3.18E-05	2.04E-03	0.803
5	Ptf1a	Pancreas-specific transcription factor, 1a	[155]	Ad	10.1	12.0	1.99E-04	5.83E-03	0.849
6	Slc9a4	Solute carrier family 9, subfamily A (NHE4, cation proton antiporter 4), member 4	[156]	Lu	59.4	64.8	3.05E-04	7.22E-03	0.917
7	Slc9b2	Solute carrier family 9, subfamily B (NHA2, cation proton antiporter 2), member 2	-	Sp	18.4	22.4	4.37E-04	8.55E-03	0.820
8	Adam28	ADAM metallopeptidase domain 28	[157]	Ad	44.4	46.2	5.68E-04	9.90E-03	0.963
9	Macc1	Metastasis associated in colon cancer 1	[158]	Li	8.7	10.1	9.53E-04	1.29E-02	0.862
10	Slc26a9	Solute carrier family 26 (anion exchanger), member 9	[159]	Ki	98.8	116.7	9.95E-04	1.32E-02	0.847
11	Psca	Prostate stem cell antigen	[160]	Co	10716.9	10801.0	1.13E-03	1.41E-02	0.992
12	Ghrl	Ghrelin/obestatin prepropeptide	[161]	Sp	1965.4	2120.2	1.63E-03	1.70E-02	0.927
13	Vsig1	V-set and immunoglobulin domain containing 1	[162]	Br	270.6	274.9	2.01E-03	1.89E-02	0.984
14	Pik3c2g	Phosphatidylinositol-4-phosphate 3-kinase, catalytic subunit type 2 gamma	-	Co	19.2	25.9	2.39E-03	2.07E-02	0.741
15	Atp4b	ATPase, H+/K+ exchanging, beta polypeptide	[163]	Du	3191.2	3201.1	2.63E-03	2.18E-02	0.997
16	Slc26a7	Solute carrier family 26 (anion exchanger), member 7	[164]	Co	17.0	19.5	2.72E-03	2.21E-02	0.876
17	Atp4a	ATPase, H+/K+ exchanging, alpha polypeptide	[163]	Ki	1945.2	1952.4	3.51E-03	2.53E-02	0.996
18	Clic6	Chloride intracellular channel 6	[165]	He	230.4	241.4	5.96E-03	3.34E-02	0.954
19	Gkn1	Gastrokine 1	[166]	Ad	58685.7	59018.3	6.44E-03	3.48E-02	0.994
20	Hdc	Histidine decarboxylase	[167]	Sp	154.9	178.9	9.09E-03	4.19E-02	0.866

**Note:** Sorted by q-value. Ad, adrenal gland; Br, brain; Co, colon; Du, duodenum; He, heart; Ki, kidney; Li, liver; Lu, lung; Sp, spleen.

* Last Ref. was based on the reports documented in PubMed (www.pubmed.gov) before June 10, 2023.

**Table 12:** Selective KEGG pathways in the adrenal gland.

**No**	**ID**	**Description**	**Significant**	**Annotated**	***p*-value**	**q-value**
1	ko03010*	Ribosome	21/283	133/5400	4.36E-06	0.001
2	ko03050	Proteasome	10/283	39/5400	2.19E-05	0.002
3	ko00061	Fatty acid biosynthesis	5/283	11/5400	1.36E-04	0.008
4	ko03020	RNA polymerase	7/283	27/5400	3.61E-04	0.014
5	ko04925*	Aldosterone synthesis and secretion	9/283	44/5400	3.67E-04	0.014
6	ko00240*	Pyrimidine metabolism	12/283	78/5400	6.58E-04	0.020

**Note:** * also significantly expressed in other organs. Sorted by q-value.

**Table 13:** Top 20 of 50 Selective KEGG pathways in the brain.

**No**	**ID**	**Description**	**Significant**	**Annotated**	***p*-value**	**q-value**
1	ko04721	Synaptic vesicle cycle	33/874	43/5400	0.000	0.000
2	ko04724	Glutamatergic synapse	39/874	67/5400	0.000	0.000
3	ko04723	Retrograde endocannabinoid signaling	38/874	65/5400	0.000	0.000
4	ko04080*	Neuroactive ligand-receptor interaction	77/874	218/5400	0.000	0.000
5	ko04727	GABAergic synapse	31/874	55/5400	0.000	0.000
6	ko04725	Cholinergic synapse	31/874	65/5400	0.000	0.000
7	ko04728	Dopaminergic synapse	36/874	87/5400	0.000	0.000
8	ko04713	Circadian entrainment	28/874	59/5400	0.000	0.000
9	ko04360*	Axon guidance	44/874	118/5400	0.000	0.000
10	ko04020*	Calcium signaling pathway	39/874	105/5400	0.000	0.000
11	ko04726	Serotonergic synapse	30/874	73/5400	0.000	0.000
12	ko04911	Insulin secretion	24/874	53/5400	0.000	0.000
13	ko04921	Oxytocin signaling pathway	36/874	99/5400	0.000	0.000
14	ko04024	cAMP signaling pathway	40/874	117/5400	0.000	0.000
15	ko04540*	Gap junction	26/874	63/5400	0.000	0.000
16	ko04072*	Phospholipase D signaling pathway	32/874	90/5400	0.000	0.000
17	ko04261*	Adrenergic signaling in cardiomyocytes	32/874	92/5400	0.000	0.000
18	ko04114	Oocyte meiosis	29/874	80/5400	0.000	0.000
19	ko04070	Phosphatidylinositol signaling system	23/874	58/5400	0.000	0.000
20	ko04915	Estrogen signaling pathway	23/874	60/5400	0.000	0.000

**Note:** * also significantly expressed in other organs. Sorted by q-value.

**Table 14:** Selective KEGG pathways in the colon.

**No.**	**ID**	**Description**	**Significant**	**Annotated**	***p*-value**	**q-value**
1	ko04630	Jak-STAT signaling pathway	19/218	94/5400	3.69E-09	5.12E-07
2	ko04060*	Cytokine-cytokine receptor interaction	23/218	163/5400	1.07E-07	7.46E-06
3	ko04064*	NF-kappa B signaling pathway	12/218	65/5400	8.39E-06	0.000
4	ko04380*	Osteoclast differentiation	13/218	87/5400	3.82E-05	0.001
5	ko04210	Apoptosis	14/218	102/5400	5.09E-05	0.001
6	ko04672*	Intestinal immune network for IgA production	7/218	32/5400	2.25E-04	0.005
7	ko04660*	T cell receptor signaling pathway	11/218	78/5400	2.58E-04	0.005
8	ko04071*	Sphingolipid signaling pathway	11/218	85/5400	5.52E-04	0.010
9	ko04214	Apoptosis - fly	7/218	43/5400	1.48E-03	0.021
10	ko04620*	Toll-like receptor signaling pathway	9/218	68/5400	1.49E-03	0.021
11	ko04919*	Thyroid hormone signaling pathway	9/218	69/5400	1.66E-03	0.021
12	ko04621*	NOD-like receptor signaling pathway	7/218	45/5400	1.94E-03	0.023
13	ko04520*	Adherens junction	7/218	46/5400	2.22E-03	0.024
14	ko04068*	FoxO signaling pathway	10/218	94/5400	4.34E-03	0.043

**Note:** * also significantly expressed in other organs. Sorted by q-value.

**Table 15:** Selective KEGG pathways in the duodenum.

**No.**	**ID**	**Description**	**Significant**	**Annotated**	***p*-value**	**q-value**
1	ko03010*	Ribosome	29/264	133/5400	3.76E-12	6.37E-10
2	ko04975	Fat digestion and absorption	10/264	26/5400	1.75E-07	1.48E-05
3	ko04978*	Mineral absorption	10/264	30/5400	8.30E-07	4.69E-05
4	ko04974	Protein digestion and absorption	13/264	60/5400	4.44E-06	0.000
5	ko04972	Pancreatic secretion	13/264	64/5400	9.47E-06	0.000
6	ko00564	Glycerophospholipid metabolism	11/264	69/5400	0.000	0.013
7	ko00450	Selenocompound metabolism	4/264	10/5400	0.001	0.021
8	ko00561	Glycerolipid metabolism	8/264	44/5400	0.001	0.021
9	ko04141*	Protein processing in the endoplasmic reticulum	15/264	126/5400	0.001	0.021
10	ko00051	Fructose and mannose metabolism	6/264	28/5400	0.002	0.033

**Note:** * also significantly expressed in other organs. Sorted by q-value.

**Table 16:** Top 20 of 21 Selective KEGG pathways in the heart.

**No.**	**ID**	**Description**	**Significant**	**Annotated**	***p*-value**	**q-value**
1	ko00190	Oxidative phosphorylation	74/331	108/5400	1.14E-66	2.01E-64
2	ko04260	Cardiac muscle contraction	27/331	54/5400	2.79E-19	2.46E-17
3	ko00020	Citrate cycle (TCA cycle)	16/331	24/5400	1.31E-14	7.70E-13
4	ko01200*	Carbon metabolism	28/331	94/5400	5.94E-13	2.61E-11
5	ko00640*	Propanoate metabolism	9/331	21/5400	1.67E-06	5.88E-05
6	ko00620	Pyruvate metabolism	10/331	28/5400	3.18E-06	9.32E-05
7	ko01210	2-Oxocarboxylic acid metabolism	6/331	13/5400	6.02E-05	0.002
8	ko00010	Glycolysis / Gluconeogenesis	11/331	48/5400	0.000	0.002
9	ko02020	Two-component system	5/331	10/5400	0.000	0.003
10	ko00280*	Valine, leucine and isoleucine degradation	9/331	35/5400	0.000	0.003
11	ko00720	Carbon fixation pathways in prokaryotes	5/331	11/5400	0.000	0.005
12	ko04020*	Calcium signaling pathway	16/331	105/5400	0.001	0.008
13	ko04922*	Glucagon signaling pathway	11/331	59/5400	0.001	0.010
14	ko03010*	Ribosome	18/331	133/5400	0.001	0.014
15	ko04261	Adrenergic signaling in cardiomyocytes	14/331	92/5400	0.001	0.015
16	ko00650*	Butanoate metabolism	6/331	22/5400	0.002	0.017
17	ko00710	Carbon fixation in photosynthetic organisms	6/331	22/5400	0.002	0.017
18	ko00071*	Fatty acid degradation	7/331	30/5400	0.002	0.018
19	ko04022*	cGMP - PKG signaling pathway	15/331	108/5400	0.002	0.021
20	ko01230*	Biosynthesis of amino acids	10/331	63/5400	0.005	0.040

**Note:** * also significantly expressed in other organs. Sorted by q-value.

**Table 17:** Selective KEGG pathways in the ileum.

**No.**	**ID**	**Description**	**Significant**	**Annotated**	***p*-value**	**q-value**
1	ko04612	Antigen processing and presentation	20/333	63/5400	4.25E-10	7.87E-08
2	ko04144*	Endocytosis	35/333	189/5400	2.23E-09	2.06E-07
3	ko04141*	Protein processing in endoplasmic reticulum	22/333	126/5400	6.72E-06	0.000
4	ko04145*	Phagosome	21/333	121/5400	1.20E-05	0.001
5	ko03010	Ribosome	22/333	133/5400	1.65E-05	0.001
6	ko04672*	Intestinal immune network for IgA production	9/333	32/5400	9.15E-05	0.003
7	ko04514*	Cell adhesion molecules (CAMs)	17/333	108/5400	0.000	0.007
8	ko00520	Amino sugar and nucleotide sugar metabolism	9/333	37/5400	0.000	0.007

**Note:** * also significantly expressed in other organs. Sorted by q-value.

**Table 18:** Top 20 of 23 selective KEGG pathways in the kidney.

**No.**	**ID**	**Description**	**Significant**	**Annotated**	***p*-value**	**q-value**
1	ko04146*	Peroxisome	23/386	62/5400	1.04E-11	1.93E-09
2	ko04961*	Endocrine and other factor-regulated calcium reabsorption	13/386	35/5400	3.61E-07	3.35E-05
3	ko04964	Proximal tubule bicarbonate reclamation	8/386	16/5400	4.91E-06	0.000
4	ko00630*	Glyoxylate and dicarboxylate metabolism	9/386	21/5400	6.03E-06	0.000
5	ko00770	Pantothenate and CoA biosynthesis	7/386	13/5400	1.06E-05	0.000
6	ko04142*	Lysosome	18/386	87/5400	3.12E-05	0.001
7	ko00280*	Valine, leucine and isoleucine degradation	10/386	35/5400	0.000	0.003
8	ko00260*	Glycine, serine and threonine metabolism	9/386	29/5400	0.000	0.003
9	ko00071*	Fatty acid degradation	9/386	30/5400	0.000	0.003
10	ko00480	Glutathione metabolism	9/386	33/5400	0.000	0.007
11	ko00640*	Propanoate metabolism	7/386	21/5400	0.000	0.007
12	ko04614	Renin-angiotensin system	7/386	21/5400	0.000	0.007
13	ko00040*	Pentose and glucuronate interconversions	6/386	16/5400	0.001	0.008
14	ko00790	Folate biosynthesis	4/386	7/5400	0.001	0.010
15	ko00910*	Nitrogen metabolism	6/386	17/5400	0.001	0.010
16	ko01200*	Carbon metabolism	16/386	94/5400	0.001	0.010
17	ko04978*	Mineral absorption	8/386	30/5400	0.001	0.010
18	ko00330	Arginine and proline metabolism	8/386	35/5400	0.003	0.028
19	ko00730	Thiamine metabolism	3/386	5/5400	0.003	0.032
20	ko00650*	Butanoate metabolism	6/386	22/5400	0.004	0.033

**Note:** * also significantly expressed in other organs. Sorted by q-value.

**Table 19:** Top 20 of 34 selective KEGG pathways in the liver.

**No.**	**ID**	**Description**	**Significant**	**Annotated**	***p*-value**	**q-value**
1	ko04610	Complement and coagulation cascades	37/265	55/5400	1.98E-36	2.97E-34
2	ko00140	Steroid hormone biosynthesis	15/265	33/5400	7.31E-12	5.46E-10
3	ko00830	Retinol metabolism	14/265	38/5400	1.12E-09	5.59E-08
4	ko00260*	Glycine, serine and threonine metabolism	11/265	29/5400	5.09E-08	1.90E-06
5	ko03320	PPAR signaling pathway	14/265	56/5400	3.03E-07	9.05E-06
6	ko00120	Primary bile acid biosynthesis	6/265	10/5400	2.35E-06	5.85E-05
7	ko04976	Bile secretion	12/265	51/5400	4.34E-06	9.27E-05
8	ko00220	Arginine biosynthesis	6/265	12/5400	9.50E-06	0.000
9	ko00980	Metabolism of xenobiotics by cytochrome P450	9/265	32/5400	1.49E-05	0.000
10	ko01230*	Biosynthesis of amino acids	12/265	63/5400	4.30E-05	0.001
11	ko00053	Ascorbate and aldarate metabolism	5/265	10/5400	5.64E-05	0.001
12	ko00982	Drug metabolism - cytochrome P450	8/265	31/5400	8.90E-05	0.001
13	ko00340	Histidine metabolism	6/265	17/5400	0.000	0.001
14	ko01040	Biosynthesis of unsaturated fatty acids	6/265	18/5400	0.000	0.002
15	ko00591	Linoleic acid metabolism	6/265	22/5400	0.001	0.005
16	ko01200*	Carbon metabolism	13/265	94/5400	0.001	0.006
17	ko00500	Starch and sucrose metabolism	6/265	24/5400	0.001	0.007
18	ko00983	Drug metabolism - other enzymes	6/265	24/5400	0.001	0.007
19	ko00100	Steroid biosynthesis	4/265	12/5400	0.002	0.016
20	ko00430	Taurine and hypotaurine metabolism	3/265	6/5400	0.002	0.016

**Note:** * also significantly expressed in other organs. Sorted by q-value.

**Table 20:** Top 20 of 46 Selective KEGG pathways in the lung.

**No.**	**ID**	**Description**	**Significant**	**Annotated**	***p*-value**	**q-value**
1	ko04510	Focal adhesion	45/703	126/5400	4.16E-11	4.01E-09
2	ko04360*	Axon guidance	43/703	118/5400	5.36E-11	4.01E-09
3	ko04390	Hippo signaling pathway	40/703	110/5400	2.77E-10	1.38E-08
4	ko04151	PI3K-Akt signaling pathway	59/703	223/5400	2.85E-08	1.06E-06
5	ko04310	Wnt signaling pathway	34/703	100/5400	4.42E-08	1.32E-06
6	ko04550	Signaling pathways regulating pluripotency of stem cells	32/703	93/5400	8.06E-08	2.01E-06
7	ko04668*	TNF signaling pathway	29/703	83/5400	2.26E-07	4.83E-06
8	ko04392	Hippo signaling pathway - multiple species	12/703	19/5400	4.56E-07	7.60E-06
9	ko04014*	Ras signaling pathway	44/703	159/5400	4.57E-07	7.60E-06
10	ko04010*	MAPK signaling pathway	46/703	177/5400	1.76E-06	2.63E-05
11	ko04060*	Cytokine-cytokine receptor interaction	43/703	163/5400	2.52E-06	3.41E-05
12	ko04015*	Rap1 signaling pathway	37/703	132/5400	2.73E-06	3.41E-05
13	ko04062*	Chemokine signaling pathway	35/703	123/5400	3.50E-06	4.03E-05
14	ko04916*	Melanogenesis	22/703	63/5400	6.67E-06	7.12E-05
15	ko04340	Hedgehog signaling pathway	13/703	27/5400	9.61E-06	9.57E-05
16	ko04512	ECM-receptor interaction	17/703	46/5400	3.23E-05	0.000
17	ko04341	Hedgehog signaling pathway - Fly	10/703	19/5400	4.01E-05	0.000
18	ko04144*	Endocytosis	44/703	189/5400	5.91E-05	0.000
19	ko04650*	Natural killer cell mediated cytotoxicity	25/703	86/5400	5.92E-05	0.000
20	ko04810*	Regulation of actin cytoskeleton	36/703	149/5400	0.000	0.001

**Note:** * also significantly expressed in other organs. Sorted by q-value.

**Table 21:** Top 20 of 33 selective KEGG pathways in the spleen.

**No.**	**ID**	**Description**	**Significant**	**Annotated**	***p*-value**	**q-value**
1	ko04110	Cell cycle	48/667	95/5400	6.13E-20	9.87E-18
2	ko04111	Cell cycle - yeast	32/667	57/5400	2.09E-15	1.68E-13
3	ko03013	RNA transport	50/667	131/5400	2.02E-14	1.08E-12
4	ko03040	Spliceosome	44/667	113/5400	3.47E-13	1.40E-11
5	ko03030	DNA replication	20/667	29/5400	1.78E-12	5.73E-11
6	ko04064*	NF-kappa B signaling pathway	27/667	65/5400	2.64E-09	7.08E-08
7	ko04113	Meiosis - yeast	22/667	49/5400	1.43E-08	3.28E-07
8	ko03420	Nucleotide excision repair	18/667	37/5400	6.48E-08	1.30E-06
9	ko04640	Hematopoietic cell lineage	21/667	49/5400	8.29E-08	1.48E-06
10	ko03460	Fanconi anemia pathway	15/667	32/5400	1.52E-06	2.45E-05
11	ko03430	Mismatch repair	9/667	14/5400	7.15E-06	0.000
12	ko03015	mRNA surveillance pathway	23/667	73/5400	1.19E-05	0.000
13	ko04662	B cell receptor signaling pathway	17/667	47/5400	2.23E-05	0.000
14	ko04060*	Cytokine-cytokine receptor interaction	39/667	163/5400	2.50E-05	0.000
15	ko03008	Ribosome biogenesis in eukaryotes	20/667	62/5400	3.01E-05	0.000
16	ko03410	Base excision repair	12/667	28/5400	5.29E-05	0.001
17	ko04660*	T cell receptor signaling pathway	22/667	78/5400	0.000	0.001
18	ko04380*	Osteoclast differentiation	23/667	87/5400	0.000	0.002
19	ko03018	RNA degradation	18/667	61/5400	0.000	0.002
20	ko04115	p53 signaling pathway	16/667	53/5400	0.000	0.004

**Note:** * also significantly expressed in other organs. Sorted by q-value.

**Table 22:** Selective KEGG pathways in the stomach.

**No.**	**ID**	**Description**	**Significant**	**Annotated**	***p*-value**	**q-value**
1	ko04971*	Gastric acid secretion	7/117	42/5400	2.70E-05	0.003
2	ko04080*	Neuroactive ligand-receptor interaction	14/117	218/5400	0.000	0.012
3	ko04270*	Vascular smooth muscle contraction	8/117	80/5400	0.0001	0.012

**Note:** * also significantly expressed in other organs. Sorted by q-value.

**Table 23:** Top 20 of 122 selective GO pathways in the adrenal gland.

**No.**	**GO.ID**	**Term**	**Ontology**	**Significant**	**Annotated**	***p*-value**	**q-value**
1	GO:0043231*	Intracellular membrane-bounded organelle	cellular component	621/998	8545/18378	8.00E-25	1.43E-21
2	GO:0005739*	Mitochondrion	cellular component	178/998	1536/18378	3.50E-23	3.12E-20
3	GO:0044424*	Intracellular part	cellular component	782/998	11898/18378	5.40E-22	2.85E-19
4	GO:0043227*	Membrane-bounded organelle	cellular component	686/998	9971/18378	6.40E-22	2.85E-19
5	GO:0044429*	Mitochondrial part	cellular component	107/998	727/18378	1.50E-21	5.35E-19
6	GO:0043226*	Organelle	cellular component	743/998	11246/18378	7.60E-20	2.26E-17
7	GO:0005622*	Intracellular	cellular component	800/998	12452/18378	1.90E-19	4.84E-17
8	GO:0008152*	Metabolic process	biological process	677/932	10277/17378	7.90E-19	1.18E-14
9	GO:0043229*	Intracellular organelle	cellular component	690/998	10283/18378	1.20E-18	2.67E-16
10	GO:0005759*	Mitochondrial matrix	cellular component	52/998	240/18378	4.70E-18	9.31E-16
11	GO:0044237*	Cellular metabolic process	biological process	611/932	9092/17378	3.00E-17	2.25E-13
12	GO:0034660*	ncRNA metabolic process	biological process	66/932	406/17378	4.50E-16	2.25E-12
13	GO:0006807*	Nitrogen compound metabolic process	biological process	417/932	5827/17378	1.80E-13	6.75E-10
14	GO:0044422*	Organelle part	cellular component	476/998	6775/18378	4.20E-13	7.27E-11
15	GO:0071704*	Organic substance metabolic process	biological process	621/932	9627/17378	4.60E-13	1.17E-09
16	GO:0034641*	Cellular nitrogen compound metabolic process	biological process	400/932	5563/17378	4.70E-13	1.17E-09
17	GO:0005737*	Cytoplasm	cellular component	593/998	8899/18378	5.10E-13	7.27E-11
18	GO:0031974*	Membrane-enclosed lumen	cellular component	264/998	3238/18378	5.30E-13	7.27E-11
19	GO:0043233*	Organelle lumen	cellular component	264/998	3238/18378	5.30E-13	7.27E-11
20	GO:0070013*	Intracellular organelle lumen	cellular component	263/998	3235/18378	8.40E-13	1.07E-10

**Note:** * also significantly expressed in other organs. Sorted by q-value.

**Table 24:** Top 20 of 897 selective GO pathways in the brain.

**No.**	**GO.ID**	**Term**	**Ontology**	**Significant**	**Annotated**	***p*-value**	**q-value**
1	GO:0097458	Neuron part	Cellular component	569/3717	1181/18378	1.00E-30	8.49E-29
2	GO:0045202	Synapse	Cellular component	409/3717	718/18378	1.00E-30	8.49E-29
3	GO:0044456	Synapse part	Cellular component	354/3717	593/18378	1.00E-30	8.49E-29
4	GO:0043005	Neuron projection	Cellular component	428/3717	875/18378	1.00E-30	8.49E-29
5	GO:0120025	Plasma membrane-bounded cell projection	Cellular component	565/3717	1477/18378	1.00E-30	8.49E-29
6	GO:0098793	Presynapse	Cellular component	193/3717	302/18378	1.00E-30	8.49E-29
7	GO:0036477	Somatodendritic compartment	Cellular component	311/3717	639/18378	1.00E-30	8.49E-29
8	GO:0042995*	Cell projection	Cellular component	581/3717	1558/18378	1.00E-30	8.49E-29
9	GO:0097060	Synaptic membrane	Cellular component	146/3717	208/18378	1.00E-30	8.49E-29
10	GO:0098794	Postsynapse	Cellular component	204/3717	354/18378	1.00E-30	8.49E-29
11	GO:0030424	Axon	Cellular component	203/3717	360/18378	1.00E-30	8.49E-29
12	GO:0030425	Dendrite	Cellular component	222/3717	436/18378	1.00E-30	8.49E-29
13	GO:0044463*	Cell projection part	Cellular component	349/3717	860/18378	1.00E-30	8.49E-29
14	GO:0043025	Neuronal cell body	Cellular component	215/3717	437/18378	1.00E-30	8.49E-29
15	GO:0045211	Postsynaptic membrane	Cellular component	108/3717	153/18378	1.00E-30	8.49E-29
16	GO:0044297	Cell body	Cellular component	232/3717	497/18378	1.00E-30	8.49E-29
17	GO:0098984	Neuron to neuron synapse	Cellular component	110/3717	181/18378	1.00E-30	8.49E-29
18	GO:0014069	Postsynaptic density	Cellular component	107/3717	176/18378	1.00E-30	8.49E-29
19	GO:0032279	Asymmetric synapse	Cellular component	108/3717	179/18378	1.00E-30	8.49E-29
20	GO:0099572	Postsynaptic specialization	Cellular component	107/3717	177/18378	1.00E-30	8.49E-29

**Note:** * also significantly expressed in other organs. Sorted by q-value.

**Table 25:** Top 20 of 536 selective GO pathways in the colon.

**No.**	**GO.ID**	**Term**	**Ontology**	**Significant**	**Annotated**	***p*-value**	**q-value**
1	GO:0002376*	Immune system process	Biological process	153/678	1949/17378	5.70E-18	8.55E-14
2	GO:0031347*	Regulation of defense response	Biological process	52/678	407/17378	4.10E-14	3.07E-10
3	GO:0002682*	Regulation of immune system process	Biological process	89/678	1014/17378	3.80E-13	1.31E-09
4	GO:0019221*	Cytokine-mediated signaling pathway	Biological process	44/678	322/17378	3.80E-13	1.31E-09
5	GO:0045321*	Leukocyte activation	Biological process	70/678	703/17378	4.60E-13	1.31E-09
6	GO:0006952*	Defense response	Biological process	93/678	1091/17378	5.70E-13	1.31E-09
7	GO:0001775*	Cell activation	Biological process	76/678	804/17378	6.10E-13	1.31E-09
8	GO:0042110*	T cell activation	Biological process	46/678	356/17378	8.50E-13	1.50E-09
9	GO:0080134*	Regulation of response to stress	Biological process	83/678	927/17378	9.00E-13	1.50E-09
10	GO:0009607*	Response to biotic stimulus	Biological process	74/678	797/17378	3.10E-12	4.63E-09
11	GO:0009605*	Response to external stimulus	Biological process	132/678	1856/17378	3.40E-12	4.63E-09
12	GO:0006955*	Immune response	Biological process	97/678	1208/17378	5.70E-12	7.12E-09
13	GO:0048518*	Positive regulation of biological process	Biological process	258/678	4587/17378	8.20E-12	9.46E-09
14	GO:0002520*	Immune system development	Biological process	67/678	706/17378	1.40E-11	1.32E-08
15	GO:0035556*	Intracellular signal transduction	Biological process	139/678	2034/17378	1.50E-11	1.32E-08
16	GO:0071345*	Cellular response to cytokine stimulus	Biological process	55/678	518/17378	1.50E-11	1.32E-08
17	GO:0043207*	Response to external biotic stimulus	Biological process	70/678	757/17378	1.50E-11	1.32E-08
18	GO:0007159*	Leukocyte cell-cell adhesion	Biological process	37/678	268/17378	2.20E-11	1.83E-08
19	GO:0031349*	Positive regulation of defense response	Biological process	34/678	231/17378	2.40E-11	1.89E-08
20	GO:0046649*	Lymphocyte activation	Biological process	60/678	604/17378	2.70E-11	1.91E-08

**Note:** * also significantly expressed in other organs. Sorted by q-value.

**Table 26:** Top 20 of 171 selective GO pathways in the duodenum.

**No.**	**GO.ID**	**Term**	**Ontology**	**Significant**	**Annotated**	***p*-value**	**q-value**
1	GO:0042571	Immunoglobulin complex, circulating	Cellular component	74/933	98/18378	1.00E-30	2.55E-28
2	GO:0019814	Immunoglobulin complex	Cellular component	74/933	102/18378	1.00E-30	2.55E-28
3	GO:0072562*	Blood microparticle	Cellular component	78/933	173/18378	1.00E-30	2.55E-28
4	GO:0005615*	Extracellular space	Cellular component	214/933	1396/18378	1.00E-30	2.55E-28
5	GO:0044421*	Extracellular region part	Cellular component	335/933	3289/18378	1.00E-30	2.55E-28
6	GO:0005576*	Extracellular region	Cellular component	357/933	3681/18378	1.00E-30	2.55E-28
7	GO:0009897*	External side of plasma membrane	Cellular component	77/933	300/18378	1.00E-30	2.55E-28
8	GO:0006910	Phagocytosis, recognition	Biological process	74/897	108/17378	1.00E-30	5.00E-28
9	GO:0006958	Complement activation, classical pathway	Biological process	73/897	107/17378	1.00E-30	5.00E-28
10	GO:0002455	Humoral immune response mediated by circulating immunoglobulin	Biological process	73/897	115/17378	1.00E-30	5.00E-28
11	GO:0006911	Phagocytosis, engulfment	Biological process	74/897	120/17378	1.00E-30	5.00E-28
12	GO:0099024	Plasma membrane invagination	Biological process	76/897	128/17378	1.00E-30	5.00E-28
13	GO:0010324	Membrane invagination	Biological process	76/897	134/17378	1.00E-30	5.00E-28
14	GO:0006956*	Complement activation	Biological process	73/897	132/17378	1.00E-30	5.00E-28
15	GO:0050853*	B cell receptor signaling pathway	Biological process	73/897	132/17378	1.00E-30	5.00E-28
16	GO:0072376	Protein activation cascade	Biological process	73/897	143/17378	1.00E-30	5.00E-28
17	GO:0008037	Cell recognition	Biological process	80/897	182/17378	1.00E-30	5.00E-28
18	GO:0050871*	Positive regulation of B cell activation	Biological process	75/897	163/17378	1.00E-30	5.00E-28
19	GO:0002377*	Immunoglobulin production	Biological process	86/897	224/17378	1.00E-30	5.00E-28
20	GO:0006959*	Humoral immune response	Biological process	78/897	188/17378	1.00E-30	5.00E-28

**Note:** * also significantly expressed in other organs. Sorted by q-value.

**Table 27:** Top 20 of 554 selective GO pathways in the heart.

**No.**	**GO.ID**	**Term**	**Ontology**	**Significant**	**Annotated**	***p*-value**	**q-value**
1	GO:0005739*	Mitochondrion	Cellular component	348/1048	1536/18378	1.00E-30	6.85E-29
2	GO:0044429*	Mitochondrial part	Cellular component	230/1048	727/18378	1.00E-30	6.85E-29
3	GO:0005743*	Mitochondrial inner membrane	Cellular component	141/1048	367/18378	1.00E-30	6.85E-29
4	GO:0031966*	Mitochondrial membrane	Cellular component	164/1048	508/18378	1.00E-30	6.85E-29
5	GO:0005740*	Mitochondrial envelope	Cellular component	167/1048	546/18378	1.00E-30	6.85E-29
6	GO:0098800	Inner mitochondrial membrane protein complex	Cellular component	85/1048	125/18378	1.00E-30	6.85E-29
7	GO:0019866*	Organelle inner membrane	Cellular component	142/1048	409/18378	1.00E-30	6.85E-29
8	GO:0098798	Mitochondrial protein complex	Cellular component	89/1048	144/18378	1.00E-30	6.85E-29
9	GO:0044455*	Mitochondrial membrane part	Cellular component	101/1048	195/18378	1.00E-30	6.85E-29
10	GO:0031967*	Organelle envelope	Cellular component	182/1048	867/18378	1.00E-30	6.85E-29
11	GO:0031975*	Envelope	Cellular component	182/1048	869/18378	1.00E-30	6.85E-29
12	GO:0070469	Respiratory chain	Cellular component	65/1048	100/18378	1.00E-30	6.85E-29
13	GO:0098803	Respiratory chain complex	Cellular component	59/1048	85/18378	1.00E-30	6.85E-29
14	GO:0005746	Mitochondrial respiratory chain	Cellular component	58/1048	86/18378	1.00E-30	6.85E-29
15	GO:0030016	Myofibril	Cellular component	76/1048	161/18378	1.00E-30	6.85E-29
16	GO:0043292	Contractile fiber	Cellular component	76/1048	171/18378	1.00E-30	6.85E-29
17	GO:0030017	Sarcomere	Cellular component	69/1048	142/18378	1.00E-30	6.85E-29
18	GO:0044449	Contractile fiber part	Cellular component	71/1048	154/18378	1.00E-30	6.85E-29
19	GO:1990204	Oxidoreductase complex	Cellular component	59/1048	105/18378	1.00E-30	6.85E-29
20	GO:0005759*	Mitochondrial matrix	Cellular component	79/1048	240/18378	1.00E-30	6.85E-29

**Note:** * also significantly expressed in other organs. Sorted by q-value.

**Table 28:** Top 20 of 141 selective GO pathways in the ileum.

**No.**	**GO.ID**	**Term**	**Ontology**	**Significant**	**Annotated**	***p*-value**	**q-value**
1	GO:1990904*	Ribonucleoprotein complex	Cellular component	144/1063	1129/18378	5.60E-20	9.98E-17
2	GO:0030529*	Intracellular ribonucleoprotein complex	Cellular component	143/1063	1128/18378	1.30E-19	1.16E-16
3	GO:0003735*	Structural constituent of ribosome	Molecular function	80/929	507/16814	9.90E-18	4.07E-14
4	GO:0005840*	Ribosome	Cellular component	86/1063	587/18378	1.10E-15	6.53E-13
5	GO:0005198*	Structural molecule activity	Molecular function	109/929	898/16814	3.30E-15	6.79E-12
6	GO:0042611	MHC protein complex	Cellular component	16/1063	25/18378	1.80E-14	8.02E-12
7	GO:0019882	Antigen processing and presentation	Biological process	27/976	91/17378	3.70E-13	5.55E-09
8	GO:0043604*	Amide biosynthetic process	Biological process	103/976	956/17378	9.30E-11	5.00E-07
9	GO:0006412*	Translation	Biological process	97/976	881/17378	1.00E-10	5.00E-07
10	GO:0022626*	Cytosolic ribosome	Cellular component	53/1063	350/18378	1.10E-10	3.92E-08
11	GO:0043603*	Cellular amide metabolic process	Biological process	113/976	1100/17378	1.90E-10	5.50E-07
12	GO:0006518*	Peptide metabolic process	Biological process	104/976	982/17378	2.00E-10	5.50E-07
13	GO:0043043*	Peptide biosynthetic process	Biological process	97/976	893/17378	2.20E-10	5.50E-07
14	GO:0048002	Antigen processing and presentation of peptide antigen	Biological process	17/976	49/17378	5.70E-10	1.22E-06
15	GO:0022627*	Cytosolic small ribosomal subunit	Cellular component	26/1063	121/18378	4.70E-09	1.34E-06
16	GO:0044391*	Ribosomal subunit	Cellular component	58/1063	446/18378	5.80E-09	1.34E-06
17	GO:0015935*	Small ribosomal subunit	Cellular component	30/1063	158/18378	7.00E-09	1.34E-06
18	GO:0044445*	Cytosolic part	Cellular component	59/1063	460/18378	7.20E-09	1.34E-06
19	GO:0005903*	Brush border	Cellular component	23/1063	99/18378	7.50E-09	1.34E-06
20	GO:0019538*	Protein metabolic process	Biological process	372/976	5206/17378	1.20E-08	2.25E-05

**Note:** * also significantly expressed in other organs. Sorted by q-value.

**Table 29:** Top 20 of 206 selective GO pathways in the kidney.

**No.**	**GO.ID**	**Term**	**Ontology**	**Significant**	**Annotated**	***p*-value**	**q-value**
1	GO:0003824*	Catalytic activity	Molecular function	571/1203	5604/16814	4.10E-26	1.69E-22
2	GO:0044281*	Small molecule metabolic process	Biological process	218/1237	1566/17378	2.20E-23	3.30E-19
3	GO:0005739*	Mitochondrion	Cellular component	210/1275	1536/18378	8.60E-23	1.53E-19
4	GO:0006082*	Organic acid metabolic process	Biological process	136/1237	806/17378	6.90E-22	5.17E-18
5	GO:0019752*	Carboxylic acid metabolic process	Biological process	128/1237	740/17378	1.40E-21	7.00E-18
6	GO:0044710*	Single-organism metabolic process	Biological process	378/1237	3483/17378	4.80E-20	1.80E-16
7	GO:0070062*	Extracellular exosome	Cellular component	253/1275	2097/18378	7.70E-20	6.86E-17
8	GO:0043436*	Oxoacid metabolic process	Biological process	130/1237	793/17378	8.00E-20	2.40E-16
9	GO:0055114*	Oxidation-reduction process	Biological process	148/1237	967/17378	1.30E-19	3.25E-16
10	GO:1903561*	Extracellular vesicle	Cellular component	253/1275	2110/18378	1.80E-19	1.02E-16
11	GO:0043230*	Extracellular organelle	Cellular component	253/1275	2114/18378	2.30E-19	1.02E-16
12	GO:1901605*	Alpha-amino acid metabolic process	Biological process	50/1237	175/17378	5.30E-18	1.14E-14
13	GO:0016491*	Oxidoreductase activity	Molecular function	123/1203	775/16814	1.70E-17	3.50E-14
14	GO:0006520*	Cellular amino acid metabolic process	Biological process	59/1237	247/17378	6.30E-17	1.18E-13
15	GO:0044282*	Small molecule catabolic process	Biological process	54/1237	231/17378	3.60E-15	6.00E-12
16	GO:0016054*	Organic acid catabolic process	Biological process	45/1237	169/17378	4.50E-15	6.13E-12
17	GO:0046395*	Carboxylic acid catabolic process	Biological process	45/1237	169/17378	4.50E-15	6.13E-12
18	GO:0031982*	Vesicle	Cellular component	318/1275	3084/18378	9.50E-15	3.39E-12
19	GO:0048037*	Cofactor binding	Molecular function	59/1203	276/16814	1.70E-14	2.33E-11
20	GO:1901565*	Organonitrogen compound catabolic process	Biological process	47/1237	222/17378	9.80E-12	1.22E-08

**Note:** * also significantly expressed in other organs. Sorted by q-value.

**Table 30:** Top 20 of 670 selective GO pathways in the liver.

**No.**	**GO.ID**	**Term**	**Ontology**	**Significant**	**Annotated**	***p*-value**	**q-value**
1	GO:0044710*	Single-organism metabolic process	Biological process	297/630	3483/17378	1.00E-30	1.50E-27
2	GO:0043436*	Oxoacid metabolic process	Biological process	131/630	793/17378	1.00E-30	1.50E-27
3	GO:0006082*	Organic acid metabolic process	Biological process	132/630	806/17378	1.00E-30	1.50E-27
4	GO:0019752*	Carboxylic acid metabolic process	Biological process	125/630	740/17378	1.00E-30	1.50E-27
5	GO:0044281*	Small molecule metabolic process	Biological process	181/630	1566/17378	1.00E-30	1.50E-27
6	GO:0055114*	Oxidation-reduction process	Biological process	125/630	967/17378	1.00E-30	1.50E-27
7	GO:0006629*	Lipid metabolic process	Biological process	128/630	1021/17378	1.00E-30	1.50E-27
8	GO:0044712*	Single-organism catabolic process	Biological process	102/630	695/17378	1.00E-30	1.50E-27
9	GO:0044282*	Small molecule catabolic process	Biological process	57/630	231/17378	1.00E-30	1.50E-27
10	GO:0032787*	Monocarboxylic acid metabolic process	Biological process	77/630	447/17378	1.00E-30	1.50E-27
11	GO:0005615*	Extracellular space	Cellular component	146/634	1396/18378	1.00E-30	1.78E-27
12	GO:0016491*	Oxidoreductase activity	Molecular function	99/614	775/16814	1.40E-28	5.76E-25
13	GO:0003824*	Catalytic activity	Molecular function	334/614	5604/16814	7.20E-28	1.48E-24
14	GO:0008202	Steroid metabolic process	Biological process	50/630	204/17378	1.10E-27	1.50E-24
15	GO:0016054*	Organic acid catabolic process	Biological process	44/630	169/17378	1.20E-25	1.38E-22
16	GO:0046395*	Carboxylic acid catabolic process	Biological process	44/630	169/17378	1.20E-25	1.38E-22
17	GO:0044255*	Cellular lipid metabolic process	Biological process	92/630	774/17378	2.10E-24	2.25E-21
18	GO:0005576*	Extracellular region	Cellular component	234/634	3681/18378	8.70E-24	7.75E-21
19	GO:0044421*	Extracellular region part	Cellular component	214/634	3289/18378	1.20E-22	7.13E-20
20	GO:1901605*	Alpha-amino acid metabolic process	Biological process	41/630	175/17378	4.30E-22	4.30E-19

**Note:** * also significantly expressed in other organs. Sorted by q-value.

**Table 31:** Top 20 of 1389 selective GO pathways in the lung.

**No.**	**GO.ID**	**Term**	**Ontology**	**Significant**	**Annotated**	***p*-value**	**q-value**
1	GO:0072359*	Circulatory system development	Biological process	300/2858	814/17378	1.00E-30	3.49E-28
2	GO:0072358	Cardiovascular system development	Biological process	216/2858	515/17378	1.00E-30	3.49E-28
3	GO:0001944	Vasculature development	Biological process	213/2858	506/17378	1.00E-30	3.49E-28
4	GO:0048856*	Anatomical structure development	Biological process	1053/2858	4553/17378	1.00E-30	3.49E-28
5	GO:0044767*	Single-organism developmental process	Biological process	1109/2858	4861/17378	1.00E-30	3.49E-28
6	GO:0032502*	Developmental process	Biological process	1117/2858	4909/17378	1.00E-30	3.49E-28
7	GO:0001568	Blood vessel development	Biological process	204/2858	487/17378	1.00E-30	3.49E-28
8	GO:0007275*	Multicellular organism development	Biological process	969/2858	4155/17378	1.00E-30	3.49E-28
9	GO:0009653*	Anatomical structure morphogenesis	Biological process	550/2858	2009/17378	1.00E-30	3.49E-28
10	GO:0010468*	Regulation of gene expression	Biological process	828/2858	3414/17378	1.00E-30	3.49E-28
11	GO:0051252*	Regulation of RNA metabolic process	Biological process	725/2858	2910/17378	1.00E-30	3.49E-28
12	GO:0048646	Anatomical structure formation involved in morphogenesis	Biological process	281/2858	814/17378	1.00E-30	3.49E-28
13	GO:0031323*	Regulation of cellular metabolic process	Biological process	1089/2858	4879/17378	1.00E-30	3.49E-28
14	GO:0060255*	Regulation of macromolecule metabolic process	Biological process	1074/2858	4798/17378	1.00E-30	3.49E-28
15	GO:0044707*	Single-multicellular organism process	Biological process	1102/2858	4954/17378	1.00E-30	3.49E-28
16	GO:2001141*	Regulation of RNA biosynthetic process	Biological process	699/2858	2797/17378	1.00E-30	3.49E-28
17	GO:1903506*	Regulation of nucleic acid-templated transcription	Biological process	698/2858	2792/17378	1.00E-30	3.49E-28
18	GO:0019222*	Regulation of metabolic process	Biological process	1141/2858	5184/17378	1.00E-30	3.49E-28
19	GO:0006355*	Regulation of transcription, DNA-templated	Biological process	690/2858	2762/17378	1.00E-30	3.49E-28
20	GO:0019219*	Regulation of nucleobase-containing compound metabolic process	Biological process	782/2858	3239/17378	1.00E-30	3.49E-28

**Note:** * also significantly expressed in other organs. Sorted by q-value.

**Table 32:** Top 20 of 1168 selective GO pathways in the spleen.

**No.**	**GO.ID**	**Term**	**Ontology**	**Significant**	**Annotated**	***p*-value**	**q-value**
1	GO:0044428*	Nuclear part	Cellular component	851/2479	3315/18378	1.00E-30	7.43E-29
2	GO:0005634*	Nucleus	Cellular component	1216/2479	5641/18378	1.00E-30	7.43E-29
3	GO:0031981*	Nuclear lumen	Cellular component	764/2479	2915/18378	1.00E-30	7.43E-29
4	GO:0005694*	Chromosome	Cellular component	327/2479	812/18378	1.00E-30	7.43E-29
5	GO:0044427*	Chromosomal part	Cellular component	297/2479	738/18378	1.00E-30	7.43E-29
6	GO:0070013*	Intracellular organelle lumen	Cellular component	781/2479	3235/18378	1.00E-30	7.43E-29
7	GO:0031974*	Membrane-enclosed lumen	Cellular component	781/2479	3238/18378	1.00E-30	7.43E-29
8	GO:0043233*	Organelle lumen	Cellular component	781/2479	3238/18378	1.00E-30	7.43E-29
9	GO:0043228*	Non-membrane-bounded organelle	Cellular component	814/2479	3656/18378	1.00E-30	7.43E-29
10	GO:0043232*	Intracellular non-membrane-bounded organelle	Cellular component	814/2479	3656/18378	1.00E-30	7.43E-29
11	GO:0005654*	Nucleoplasm	Cellular component	554/2479	2197/18378	1.00E-30	7.43E-29
12	GO:0098687	Chromosomal region	Cellular component	136/2479	250/18378	1.00E-30	7.43E-29
13	GO:0032991*	Macromolecular complex	Cellular component	940/2479	4830/18378	1.00E-30	7.43E-29
14	GO:0000228	Nuclear chromosome	Cellular component	177/2479	457/18378	1.00E-30	7.43E-29
15	GO:0044446*	Intracellular organelle part	Cellular component	1193/2479	6591/18378	1.00E-30	7.43E-29
16	GO:0044454	Nuclear chromosome part	Cellular component	167/2479	429/18378	1.00E-30	7.43E-29
17	GO:0044422*	Organelle part	Cellular component	1204/2479	6775/18378	1.00E-30	7.43E-29
18	GO:0000775	Chromosome, centromeric region	Cellular component	83/2479	142/18378	1.00E-30	7.43E-29
19	GO:0005622*	Intracellular	Cellular component	1939/2479	12452/18378	1.00E-30	7.43E-29
20	GO:0000793	Condensed chromosome	Cellular component	82/2479	145/18378	1.00E-30	7.43E-29

**Note:** * also significantly expressed in other organs. Sorted by q-value.

**Table 33:** Top 20 of 21 selective GO pathways in the stomach.

**No.**	**GO.ID**	**Term**	**Ontology**	**Significant**	**Annotated**	***p*-value**	**q-value**
1	GO:0007586*	Digestion	Biological process	20/490	111/17378	3.40E-11	5.10E-07
2	GO:0001696	Gastric acid secretion	Biological process	9/490	17/17378	2.10E-10	1.57E-06
3	GO:0055123*	Digestive system development	Biological process	17/490	128/17378	1.20E-07	0.000
4	GO:0022600	Digestive system process	Biological process	14/490	86/17378	1.20E-07	0.000
5	GO:0031016	Pancreas development	Biological process	12/490	71/17378	6.20E-07	0.002
6	GO:0004190	Aspartic-type endopeptidase activity	Molecular function	7/487	23/16814	2.70E-06	0.006
7	GO:0070001	Aspartic-type peptidase activity	Molecular function	7/487	24/16814	3.70E-06	0.006
8	GO:0001228*	Transcriptional activator activity, RNA polymerase II transcription regulatory region sequence-specific	Molecular function	25/487	315/16814	5.50E-06	0.006
9	GO:0000981*	RNA polymerase II transcription factor activity, sequence-specific DNA binding	Molecular function	38/487	601/16814	5.80E-06	0.006
10	GO:0030855*	Epithelial cell differentiation	Biological process	33/490	488/17378	3.50E-06	0.009
11	GO:0046903*	Secretion	Biological process	49/490	879/17378	4.20E-06	0.009
12	GO:0046717*	Acid secretion	Biological process	12/490	87/17378	5.70E-06	0.011
13	GO:0031018	Endocrine pancreas development	Biological process	8/490	40/17378	1.30E-05	0.021
14	GO:0044765*	Single-organism transport	Biological process	99/490	2326/17378	1.40E-05	0.021
15	GO:0009888*	Tissue development	Biological process	69/490	1472/17378	1.80E-05	0.025
16	GO:0048565*	Digestive tract development	Biological process	13/490	117/17378	2.60E-05	0.032
17	GO:0005882	Intermediate filament	Cellular component	15/533	144/18378	1.90E-05	0.034
18	GO:0051050*	Positive regulation of transport	Biological process	43/490	793/17378	3.20E-05	0.037
19	GO:1903011	Negative regulation of bone development	Biological process	4/490	8/17378	4.00E-05	0.043
20	GO:0060428	Lung epithelium development	Biological process	7/490	35/17378	4.60E-05	0.046

**Note:** * also significantly expressed in other organs. Sorted by q-value.

**Table 34:** The top 20/32 genes were not described but selectively expressed in the adrenal glands based on their abundance (n = 3).

**No.**	**Gene ID**	**Gene Name**	**Median Organ**	**FPKM**		***p*-value**	**q-value**	**Mean** **/total**
				**Mean**	**Total**			
1	ENSRNOG00000041608	AC123095.1	St	32.5	45.5	2.39E-05	2.00E-03	0.716
2	ENSRNOG00000055956	AABR07015078.1	St	103.8	141.0	3.62E-05	2.47E-03	0.736
3	ENSRNOG00000030291	Rn50_10_0698.6	St	871.4	1199.5	4.14E-05	2.57E-03	0.727
4	ENSRNOG00000060657	AABR07000404.1	St	14.2	19.1	1.05E-04	4.21E-03	0.742
5	ENSRNOG00000029145	AY172581.2	St	462.8	594.1	1.68E-04	5.34E-03	0.779
6	ENSRNOG00000057514	AABR07015080.1	St	26.2	35.7	1.79E-04	5.51E-03	0.734
7	ENSRNOG00000057811	AABR07015055.2	St	18.8	25.1	2.46E-04	6.47E-03	0.750
8	ENSRNOG00000055836	AABR07000402.1	St	30.7	42.9	3.34E-04	7.56E-03	0.717
9	ENSRNOG00000046600	AABR07015066.1	St	73.6	100.6	3.88E-04	8.16E-03	0.732
10	ENSRNOG00000055323	AABR07063421.1	St	33.9	47.6	4.49E-04	8.79E-03	0.712
11	ENSRNOG00000046081	AABR07015079.1	St	38.8	55.2	5.83E-04	1.00E-02	0.703
12	ENSRNOG00000047991	AABR07072283.1	St	125.3	143.0	1.02E-03	1.34E-02	0.876
13	ENSRNOG00000053717	Metazoa_SRP	Il	121.7	171.4	1.69E-03	1.68E-02	0.710
14	ENSRNOG00000046768	AC135454.2	St	13.2	14.2	1.62E-03	1.70E-02	0.929
15	ENSRNOG00000056945	LOC102549408	Sp	22.1	26.9	1.97E-03	1.88E-02	0.820
16	ENSRNOG00000049380	Rn50_11_0375.8	Du	55.9	68.7	2.24E-03	1.99E-02	0.814
17	ENSRNOG00000046106	rno-mir-351-1	St	6.4	6.4	2.49E-03	2.11E-02	1.000
18	ENSRNOG00000055947	7SK	Du	24.6	30.8	2.63E-03	2.14E-02	0.800
19	ENSRNOG00000048598	AABR07037925.1	St	84.9	95.4	2.55E-03	2.14E-02	0.889
20	ENSRNOG00000053888	5_8S_rRNA	St	11.8	13.7	2.75E-03	2.23E-02	0.858

**Note:** Sorted by q-value. Du, duodenum; Il, ileum; Sp, spleen; St, stomach.

**Table 35:** The top 20/27 genes were not described but selectively expressed in the brain based on their abundance (n = 3).

**No.**	**Gene ID**	**Gene Name**	**Median Organ**	**FPKM**		***p*-value**	**q-value**	**Mean/** **total**
				**Mean**	**Total**			
1	ENSRNOG00000047491	AABR07037520.1	St	53.6	59.3	6.32E-07	3.22E-04	0.905
2	ENSRNOG00000051341	Rn50_X_0635.2	Co	28.1	35.5	3.05E-06	5.69E-04	0.792
3	ENSRNOG00000054414	AABR07043276.1	Il	23.4	28.2	4.13E-06	8.22E-04	0.831
4	ENSRNOG00000060837	AC132752.2	Il	58.1	76.0	1.22E-05	1.06E-03	0.764
5	ENSRNOG00000003025	Rn50_X_0749.3	Ad	48.1	52.7	4.53E-05	2.54E-03	0.914
6	ENSRNOG00000060863	AABR07017145.1	Sp	50.4	62.3	5.12E-05	2.92E-03	0.810
7	ENSRNOG00000060211	AABR07058699.2	Co	31.8	36.6	1.24E-04	4.36E-03	0.869
8	ENSRNOG00000054809	AABR07026032.1	Lu	8.5	8.9	1.34E-04	4.74E-03	0.954
9	ENSRNOG00000038087	AC110846.1	Li	7.7	10.3	4.35E-04	5.50E-03	0.746
10	ENSRNOG00000058047	AABR07000733.1	Il	10.3	11.2	2.19E-04	5.93E-03	0.918
11	ENSRNOG00000022286	Rn50_X_0746.6	Ad	16.9	20.2	2.85E-04	6.70E-03	0.834
12	ENSRNOG00000022267	Rn50_X_0747.1	Il	50.2	60.8	5.73E-04	9.89E-03	0.827
13	ENSRNOG00000059081	AABR07026032.3	Co	47.8	49.1	6.06E-04	1.02E-02	0.974
14	ENSRNOG00000054155	Rn50_5_1638.1	Ad	10.7	10.9	6.29E-04	1.04E-02	0.983
15	ENSRNOG00000052831	AABR07040629.1	Co	31.1	35.4	1.67E-03	1.72E-02	0.879
16	ENSRNOG00000049802	AABR07031533.1	Ki	36.4	37.0	2.35E-03	2.05E-02	0.984
17	ENSRNOG00000002734	AABR07042077.1	Il	8.2	8.4	2.83E-03	2.26E-02	0.984
18	ENSRNOG00000054121	AABR07061178.1	Du	21.5	22.4	4.28E-03	2.80E-02	0.960
19	ENSRNOG00000060858	AABR07043711.1	Ki	12.4	13.3	4.82E-03	2.95E-02	0.934
20	ENSRNOG00000058276	AABR07043200.1	Sp	7.5	8.5	5.24E-03	3.12E-02	0.887

**Note:** Sorted by q-value. Ad, adrenal gland; Co, colon; Du, duodenum; Il, ileum; Ki, kidney; Li, liver; Lu, lung; Sp, spleen; St, stomach.

**Table 36:** Genes were not described but selectively expressed in the colon based on their abundance (n = 3).

**No.**	**Gene ID**	**Gene Name**	**Median Organ**	**FPKM**		***p*-value**	**q-value**	**Mean/** **total**
				**Mean**	**Total**			
1	ENSRNOG00000062185	Rn60_20_0141.5	Ad	16.4	19.8	1.68E-05	1.68E-03	0.828
2	ENSRNOG00000056727	AABR07057353.2	St	11.5	11.7	4.92E-04	9.21E-03	0.979
3	ENSRNOG00000038598	AABR07032503.1	Ad	10.8	13.0	6.59E-03	3.52E-02	0.827

**Note:** Sorted by q-value. Ad, adrenal gland; St, stomach.

**Table 37:** Genes were not described but selectively expressed in the duodenum based on their abundance (n = 3).

**No.**	**Gene ID**	**Gene Name**	**Median Organ**	**FPKM**		***p*-value**	**q-value**	**Mean/** **total**
				**Mean**	**Total**			
1	ENSRNOG00000055064	LOC102551636	Ki	415.4	557.4	5.88E-06	9.90E-04	0.745
2	ENSRNOG00000056733	AABR07004539.1	Ad	114.7	122.9	1.02E-03	1.33E-02	0.933
3	ENSRNOG00000058562	AABR07065651.7	Br	52.1	73.9	7.41E-03	3.74E-02	0.705

**Note:** Sorted by q-value. Ad, adrenal gland; Br, brain; Ki, kidney.

**Table 38:** Genes were not described but selectively expressed in the heart based on their abundance (n = 3).

**No.**	**Gene ID**	**Gene Name**	**Median Organ**	**FPKM**		***p*-value**	**q-value**	**Mean/** **total**
				**Mean**	**Total**			
1	ENSRNOG00000023227	AABR07052585.2	Li	745.4	754.6	6.97E-07	3.41E-04	0.988
2	ENSRNOG00000043057	AABR07025284.1	Il	18.5	20.0	1.58E-06	5.10E-04	0.924
3	ENSRNOG00000052518	AABR07025387.1	Du	26.7	33.3	2.28E-05	1.89E-03	0.801
4	ENSRNOG00000048644	AC115371.1	St	13.2	13.6	1.22E-04	4.56E-03	0.970
5	ENSRNOG00000046133	LOC102553613	Du	30.7	37.8	1.67E-04	4.59E-03	0.811
6	ENSRNOG00000052389	AABR07031489.1	Co	8.9	9.9	3.03E-04	7.12E-03	0.902
7	ENSRNOG00000055328	AABR07017268.1	Ki	10.3	11.7	8.42E-04	1.20E-02	0.881
8	ENSRNOG00000060690	AABR07052523.2	Ad	8.1	8.1	1.40E-03	1.57E-02	1.000
9	ENSRNOG00000046229	AC130940.1	St	11.9	12.7	2.82E-03	2.26E-02	0.935
10	ENSRNOG00000058414	LOC103690078	Ad	5.6	5.6	1.13E-02	4.71E-02	0.992

**Note:** Sorted by q-value. Ad, adrenal gland; Co, colon; Du, duodenum; Il, ileum; Ki, kidney; Li, liver; St, stomach.

**Table 39:** Genes were not described but selectively expressed in the ileum based on their abundance (n = 3).

**No.**	**Gene ID**	**Gene Name**	**Median Organ**	**FPKM**		***p-*value**	**q-value**	**Mean/** **total**
				**Mean**	**Total**			
1	ENSRNOG00000051194	LOC108352134	Ad	22.7	30.7	4.35E-03	2.83E-02	0.739
2	ENSRNOG00000051320	Rn50_7_1164.3	Lu	22.0	25.6	4.74E-03	2.95E-02	0.861

**Note:** Sorted by q-value. Ad, adrenal gland; Lu, lung.

**Table 40:** Genes were not described but selectively expressed in the kidney based on their abundance (n = 3).

**No.**	**Gene ID**	**Gene Name**	**Median Organ**	**FPKM**		***p*-value**	**q-value**	**Mean/** **total**
				**Mean**	**Total**			
1	ENSRNOG00000056396	AABR07006120.1	Ad	29.7	29.7	1.76E-06	5.42E-04	1.000
2	ENSRNOG00000054801	AABR07057997.1	Br	8.2	8.9	2.50E-06	6.28E-04	0.926
3	ENSRNOG00000051964	LOC103691699	St	67.9	75.7	6.31E-06	9.80E-04	0.897
4	ENSRNOG00000054733	LOC103690137	He	10.9	14.5	1.63E-05	1.11E-03	0.756
5	ENSRNOG00000061754	LOC102555924	Sp	5.5	7.2	1.78E-05	1.36E-03	0.762
6	ENSRNOG00000057101	AABR07050652.1	Sp	15.5	20.5	4.17E-05	2.47E-03	0.759
7	ENSRNOG00000057904	LOC102554608	Ad	64.2	64.2	2.14E-04	6.03E-03	1.000
8	ENSRNOG00000061966	Rn60_1_2220.2	Ad	15.2	17.1	2.46E-04	6.48E-03	0.891
9	ENSRNOG00000061127	AABR07057844.2	He	8.8	9.4	2.89E-04	6.98E-03	0.936
10	ENSRNOG00000061436	AABR07026778.1	Ad	27.7	27.8	4.18E-04	8.48E-03	0.997
11	ENSRNOG00000059212	AABR07025303.1	Lu	14.2	15.9	6.33E-04	1.03E-02	0.895
12	ENSRNOG00000053953	AABR07016672.1	Ad	6.9	6.9	9.28E-04	1.27E-02	1.000
13	ENSRNOG00000057369	AABR07027240.1	Ad	7.8	7.8	1.00E-03	1.32E-02	0.997
14	ENSRNOG00000059314	AABR07013477.2	Ad	16.7	16.8	1.14E-03	1.41E-02	0.994
15	ENSRNOG00000046343	-	Ad	47.6	57.0	1.23E-03	1.47E-02	0.835
16	ENSRNOG00000058847	AABR07044001.4	Br	10.6	14.9	1.84E-03	1.80E-02	0.710
17	ENSRNOG00000058611	AABR07027137.1	Lu	14.2	19.6	9.08E-03	4.14E-02	0.723

**Note:** Sorted by q-value. Ad, adrenal gland; Br, brain; He, heart; Lu, lung; Sp, spleen; St, stomach.

**Table 41:** Genes were not described but selectively expressed in the liver based on their abundance (n = 3).

**No.**	**Gene ID**	**Gene Name**	**Median Organ**	**FPKM**		***p*-value**	**q-value**	**Mean/** **total**
				**Mean**	**Total**			
1	ENSRNOG00000054077	AABR07024870.1	Ad	277.7	277.8	8.88E-05	3.87E-03	1.000
2	ENSRNOG00000052176	AC115255.1	Du	9.3	10.4	3.78E-04	7.88E-03	0.895
3	ENSRNOG00000059330	AABR07004549.1	Ad	802.9	803.1	1.56E-03	1.66E-02	1.000
4	ENSRNOG00000062027	Rn60_12_0107.3	Ad	89.4	89.5	1.73E-03	1.75E-02	0.999
5	ENSRNOG00000021575	AABR07021096.1	Ad	42.3	42.9	4.88E-03	3.01E-02	0.987
6	ENSRNOG00000055973	AABR07058498.1	Ad	14.5	14.9	5.54E-03	3.22E-02	0.975

**Note:** Sorted by q-value. Ad, adrenal gland; Du, duodenum

**Table 42:** Genes were not described but selectively expressed in the lung based on their abundance (n = 3).

**No.**	**Gene ID**	**Gene Name**	**Median Organ**	**FPKM**		***p*-value**	**q-value**	**Mean/** **total**
				**Mean**	**Total**			
1	ENSRNOG00000054709	AABR07061382.2	St	14.2	18.3	2.75E-06	3.69E-04	0.776
2	ENSRNOG00000053542	AABR07067469.1	Ad	11.5	11.9	1.45E-05	1.56E-03	0.963
3	ENSRNOG00000055889	AABR07030901.1	He	5.7	7.2	6.63E-05	3.16E-03	0.792
4	ENSRNOG00000036872	AC119007.1	St	29.1	30.6	1.51E-04	5.07E-03	0.950
5	ENSRNOG00000059588	AC113785.2	Ki	1016.6	1365.0	2.14E-04	6.04E-03	0.745
6	ENSRNOG00000046001	AABR07030823.1	He	22.4	28.4	8.07E-04	1.16E-02	0.790
7	ENSRNOG00000052597	AABR07062477.2	Ad	7.0	7.0	8.03E-04	1.18E-02	0.995
8	ENSRNOG00000050974	AABR07030773.1	St	9.3	12.2	3.21E-03	2.39E-02	0.761
9	ENSRNOG00000054935	Rn50_7_1408.2	St	14.7	15.0	5.45E-03	3.19E-02	0.980

**Note:** Sorted by q-value. Ad, adrenal gland; He, heart; Ki, kidney; St, stomach.

**Table 43:** Genes were not described but selectively expressed in the spleen based on their abundance (n = 3).

**No.**	**Gene ID**	**Gene Name**	**Median Organ**	**FPKM**		***p*-value**	**q-value**	**Mean/** **total**
				**Mean**	**Total**			
1	ENSRNOG00000062220	LOC679342	St	9.1	12.0	3.88E-07	2.54E-04	0.764
2	ENSRNOG00000062144	AABR07035955.1	St	34.0	45.8	4.49E-06	8.67E-04	0.742
3	ENSRNOG00000053879	AABR07071821.1	Ad	8.8	8.9	5.70E-05	3.10E-03	0.988
4	ENSRNOG00000060395	AABR07025301.1	St	10.4	13.4	1.30E-04	4.70E-03	0.780
5	ENSRNOG00000057558	AC128792.2	Ki	1492.9	1879.3	1.89E-04	5.62E-03	0.794
6	ENSRNOG00000053143	Rn50_7_1407.3	Du	17.7	20.4	1.35E-03	1.41E-02	0.866
7	ENSRNOG00000041826	AABR07053152.1	St	14.6	19.8	1.66E-03	1.71E-02	0.736
8	ENSRNOG00000041746	AC095678.1	St	6.1	7.3	1.86E-03	1.82E-02	0.832
9	ENSRNOG00000039025	AABR07051947.1	Lu	24.8	34.8	4.04E-03	2.66E-02	0.713
10	ENSRNOG00000052921	AABR07021221.1	Ki	19.8	25.1	9.51E-03	4.25E-02	0.788
11	ENSRNOG00000054411	AABR07072897.1	St	6.4	8.9	1.07E-02	4.58E-02	0.714
12	ENSRNOG00000062261	Rn60_15_0518.2	Ad	6.5	6.7	1.18E-02	4.82E-02	0.977

**Note:** Sorted by q-value. Ad, adrenal gland; Du, duodenum; Ki, kidney; Lu, lung; St, stomach.

**Table 44:** Genes were not described but selectively expressed in the stomach based on their abundance (n = 3).

**No.**	**Gene ID**	**Gene Name**	**Median Organ**	**FPKM**		***p*-value**	**q-value**	**Mean/** **total**
				**Mean**	**Total**			
1	ENSRNOG00000060525	AABR07007717.3	Du	8.9	12.7	5.70E-05	2.11E-03	0.703
2	ENSRNOG00000062012	Rn60_20_0037.1	Ad	35.3	35.3	6.37E-03	3.46E-02	1.000

**Note:** Sorted by q-value. Ad, adrenal gland; Du, duodenum.

## Data Availability

The raw data were uploaded as supplemental materials on the journal’s web.

## LIST OF ABBREVIATIONS

DOS: Disc Operation System
GO: Gene Ontology
Icam1: Intercellular Adhesion Molecule 1
KEGG: Kyoto Encyclopedia of Genes and Genomes
NGAL: Neutrophil Gelatinase-associated Lipocalin
PDE5a: Phosphodiesterase 5
SD: Sprague-Dawley

## SUPPLEMENTARY MATERIAL

Supplementary material is available on the publisher’s website along with the published article. The raw data were uploaded on July 19, 2023 (Link: https://pan.baidu.com/s/1uOpvEIU_dRYgGmEIWc0SjA?pwd=DWG1 Password: DWG1)
